# Comparing somatic mutation-callers: beyond Venn diagrams

**DOI:** 10.1186/1471-2105-14-189

**Published:** 2013-06-10

**Authors:** Su Yeon Kim, Terence P Speed

**Affiliations:** 1 Department of Statistics, University of California at Berkeley, 367 Evans Hall, Berkeley, CA 94720 USA; 2 Walter and Eliza Hall Institute of Medical Research, Parkville, Victoria, Australia

**Keywords:** Cancer genome, Next-generation sequencing, Somatic mutation-calling, Methods comparison, Validation

## Abstract

**Background:**

Somatic mutation-calling based on DNA from matched tumor-normal patient samples is one of the key tasks carried by many cancer genome projects. One such large-scale project is The Cancer Genome Atlas (TCGA), which is now routinely compiling catalogs of somatic mutations from hundreds of paired tumor-normal DNA exome-sequence data. Nonetheless, mutation calling is still very challenging. TCGA benchmark studies revealed that even relatively recent mutation callers from major centers showed substantial discrepancies. Evaluation of the mutation callers or understanding the sources of discrepancies is not straightforward, since for most tumor studies, validation data based on independent whole-exome DNA sequencing is not available, only partial validation data for a selected (ascertained) subset of sites.

**Results:**

To provide guidelines to comparing outputs from multiple callers, we have analyzed two sets of mutation-calling data from the TCGA benchmark studies and their partial validation data. Various aspects of the mutation-calling outputs were explored to characterize the discrepancies in detail. To assess the performances of multiple callers, we introduce four approaches utilizing the external sequence data to varying degrees, ranging from having independent DNA-seq pairs, RNA-seq for tumor samples only, the original exome-seq pairs only, or none of those.

**Conclusions:**

Our analyses provide guidelines to visualizing and understanding the discrepancies among the outputs from multiple callers. Furthermore, applying the four evaluation approaches to the whole exome data, we illustrate the challenges and highlight the various circumstances that require extra caution in assessing the performances of multiple callers.

## Background

Cancer is driven by genomic alterations. Recent advances in high-throughput sequencing technologies allow identification of somatic alterations at an unprecedented resolution [1-4]. In particular, numerous large-scale cancer projects scan for somatic mutations in various tumor types, prior to conducting downstream analyses such as detecting significantly mutated genes or pathways [5-9], inferring clonal history [10,11], and characterizing the landscape of the somatic mutations [12-14].

Nonetheless, accurate somatic mutation-calling using high-throughput sequence data remains one of the major challenges in cancer genomics. For somatic mutation-calling, one looks for a site in which a variant allele exists in the tumor sample but not in the normal sample. Even with the sequence data from a normal sample, variant calling in high-throughput sequencing data is challenging due to various sources of errors such as artifacts occurring during PCR amplification or targeted capture (eg. exome-capture), machine sequencing errors, incorrect local alignments of reads [15-18]. Tumor heterogeneity and normal contamination add additional challenges for the tumor samples.

Understanding mutation-calling errors is constrained by the availability of the validation data. False positives can be learned by validating every called site, but experimentally validating all such sites across a whole exome or genome is laborious and expensive. Learning about false negatives can be even more difficult. Even independent sequencing at a much higher sequencing depth or using a different sequencing technology does not guarantee the finding of all true mutations. Comparing outputs from multiple callers and learning from their discrepancies, however, can be a fast and practical solution to study calling errors to some extent. False negatives from one caller could be revealed by mutations detected by other callers. Calls that are uniquely detected by a certain caller are likely to reveal at least a partial set of false positives from that caller.

One such comparison example is the The Cancer Genome Atlas mutation-calling benchmark study in which several major analysis centers called mutations on a set of selected samples. A Venn diagram summarization of the outputs immediately revealed substantial discrepancies among the calls from different centers, but a more thorough analysis was necessary for better characterization and assessments of the callers. Similar exercises can be easily conceived by anyone who is interested in performing mutation analysis, since mutation-calling is a rapidly growing field and there is unlikely to be a single “best” caller.

To provide guidelines for more illuminating analyses in comparing multiple callers beyond Venn diagrams, we present how we have analyzed two datasets generated for TCGA benchmark studies. One dataset was generated by applying four mutation-callers to Illumina exome-seq pairs from 16 lung squamous cell carcinoma (LUSC) patients. The other dataset was generated by applying three callers to SOLiD exome-seq pairs from 6 rectum adenocarcinoma (READ) patients. For each dataset, partial validation data exists. For the LUSC dataset, 76 genes were independently sequenced at a higher coverage by the Illumina technology, allowing in-depth investigation of called sites within the 76 genes. For the READ dataset, validation information based on the 454 sequencing technology was available for 721 sites.

We characterized the discrepancies in the two datasets in various ways. Furthermore, based on the insights gained by analyzing these data, we introduced four approaches to comparing the relative performances of multiple callers. The first is using independent DNA-sequencing data, available for 76 genes for LUSC patients, to validate detected mutations. The second utilizes the tumor RNA-seq data to validate variants in the tumor samples. The third method uses the variant quality scores obtained by running a publicly available variant caller, the GATK UnifiedGenotyper[19], on the original exome-seq data, to define pseudo-positives (presumed somatic mutations) and pseudo-negatives. The last approach estimates the sensitivity and the specificity of each caller from the observed counts of mutations classified by the detection status of the multiple callers, using the statistical method called latent class models [20,21].

## Results

TCGA benchmark studies generated mutation-calling outputs by applying multiple callers on the same sequence alignments (BAMs [22]) for a selected list of samples. We have analyzed two datasets, one based on lung squamous cell carcinoma (LUSC) samples and the other based on rectum adenocarcinoma (READ) samples. The callers used and the types of available sequence data are described below as well as summarized in Additional file 1: Table S1.

Note that all the mutation-calling outputs were generated before July 2011, and no current mutation-caller will be the same as those used in this study, which is a part of the reason for using anonymized callers. Even though indels and other structural variants are important kinds of somatic variants, in this paper we focus on comparing single nucleotide variant (SNV) type somatic mutations, which comprise the majority of somatic variants.

**LUSC dataset** Mutation calling was done by four callers (Caller A, B, C and D) using Illumina exome-seq tumor-normal pairs from 16 LUSC patients. Two kinds of additional data exist for the same patients. One is Illumina RNA-seq data available for the 16 tumor samples. Second is high-coverage Illumina sequencing data (∼3-fold higher coverage than the original exome-seqs of ∼80x, and thus called as ‘deep-sequencing’ data in our manuscript) available for tumor-normal pairs on a pre-selected set of 76 genes (540 Kb).

**READ dataset** Mutation calling was done by three callers (Caller H, I, and J) using SOLiD exome-seq tumor-normal pairs from 6 READ patients. Three kinds of additional data exist. One is Illumina RNA-sequence data available for the 6 tumor samples. A second is Illumina exome-seq tumor-normal pairs for all 6 patients. The last one is information available for 721 sites, for which validation was done using the 454 sequencing technology.

In an effort to create comparable calling outputs, all four centers agreed on one annotation representing exome regions and generated calls only within those regions. Outputs were provided in a modified Variant Call Format (VCF [23]), which reports the genomic position, somatic status, filter status, sequence information from each tumor and normal sample. The filter status indicates whether the variant (candidate mutation) passes all the filters implemented by each caller or not. The full details of all filters were not given in the VCF files though, partly because the modified VCF format was under active development.

Detecting a variant in an aligned sequence (BAM) is looking for the existence of a variant allele that is different from the reference allele. In principle, the more reads carrying the variant allele, the stronger the evidence for it being a true variant. Thus, the fraction of reads carrying the variant allele (called the variant allele fraction, ‘vaf’) is frequently used in variant calling analyses. For somatic mutation-calling, the tumor and its matched normal sample are considered together. Therefore, a variant is determined by the joint status in tumor-normal sequence pairs: ‘somatic’ (the variant allele is found in the tumor sample but not in the normal), ‘germline’ (variant allele found in both the tumor and the normal sample), and ‘wildtype’ (no variant allele found in either the tumor or the normal sample). In our manuscript, a mutation or variant ‘site’ refers to a position only for the particular patient carrying the variant.

### Discrepancies observed in the benchmark data

**Figure 1:**
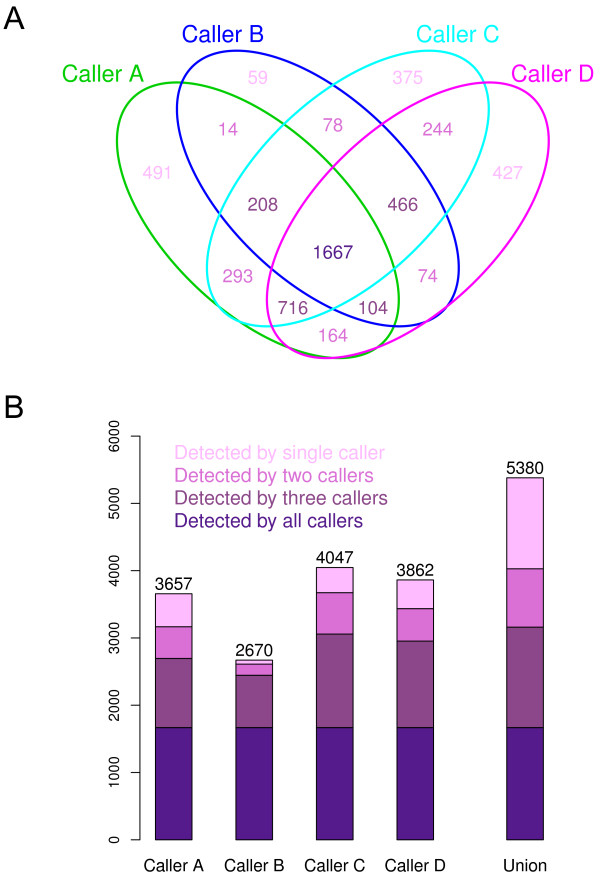
**Counts of the mutations detected by four callers in the 16 LUSC tumor-normal exome-seq pairs. A.** Venn Diagram of the mutations. **B.** Mutations detected by each caller or by any caller (‘Union’) are classified based on the number of callers detecting the mutations.

**LUSC dataset** From each caller’s raw mutation-calling output (VCF), we extracted a final set of somatic mutations. To have a broad picture, we gathered all such mutations from all 16 LUSC patients. An immediate Venn diagram summary reveals substantial discrepancies among the mutations from the four callers (Figure 1A). For example, 491 and 427 mutations were detected by Caller A only and Caller D only, while 1,667 mutations were discovered by all four callers. There are many mutations that were missed by a single caller. For example, 716 mutations were detected by all but Caller B, and 104 were detected by all but Caller C. We also categorized mutations based on the degree of agreement (Figure 1B). In total, 5,380 mutations were called by one or more callers, and 31%, 28%, 16%, and 25% of those were detected by all, three, two, and a single caller(s). A similar categorization of the mutations detected by each caller suggests that Caller B is stringent, since it detected a relatively small number of mutations, most of which were detected by the other callers. Callers A, C, and D reported a similar number of mutations, a good proportion of which are caller-specific.

**Figure 2:**
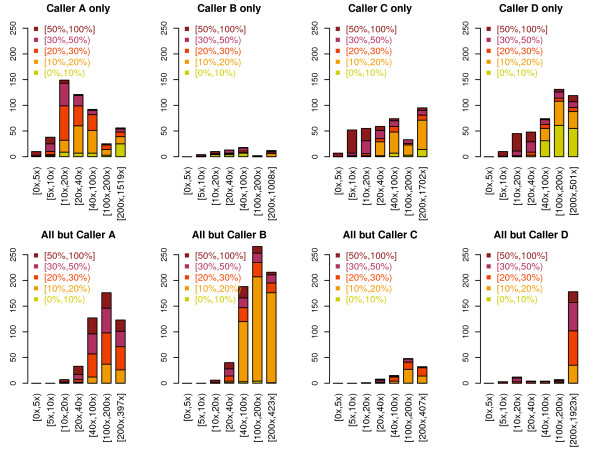
**Distribution of the coverage (horizontal) and the variant allele fraction (vertical) in the tumor exome-seqs.** Among the mutations detected by four callers using 16 LUSC tumor-normal exome-seq pairs, mutations detected by a single caller (upper row) or missed by a single caller (lower row) are used. Each column corresponds to a caller that uniquely detects the mutations or uniquely misses the mutations.

We attempted to further characterize the discrepancies among the callers using those mutations *called by a single caller only* (1,352) or *missed by a single caller only* (1,494). To a large extent, discrepancies occurred due to different variant call status on the tumor sample. Variant calling in the normal sample seems to be highly accurate, as potential mis-classification between somatic mutations and germline mutations explain relatively small fraction of the discrepancies (only 11% of the mutations have a normal vaf > 2%, which is probably a minimal requirement for a variant to be detected). The distribution of the coverages and the variant allele fractions in the tumor exome-seqs vary significantly across the mutation sets defined based on the detection status of the four callers (Figure 2). For example, the set of mutations detected by Caller A only is somewhat enriched for positions of medium- to low-sequencing depth (e.g., <40x), while the set detected by Caller D only has many sites with very high depth (>100x) but have very low vaf (<10*%*). The characteristics of mutations that were missed by a single center are different. A large fraction of mutations that were missed by Caller B only have tumor vaf <20*%*. Almost all the mutations detected by all but Caller D have very high depth (>200x), and most of them have vaf > 20%. The mutations missed by Caller A only have medium-to-high coverage (> 40x) and medium-to-high vaf > 20%. The number of mutations that were missed by Caller C is significantly lower than those missed by other callers only. Overall, many of the mutations detected by a single caller only and those missed by Caller B only have relatively low sequencing depth or low vaf, which presumably poses difficulty in detecting existence of alternative allele in the tumor sample, while mutations missed by Caller A, C, or D only tend to have high sequencing depth and show good support of the existence of a variant allele. Thus, for these mutations, it’s likely that the single caller employed a certain filter that was different from the others.

To better understand the sources of discrepancies, we examined all variants in the VCF files. (We note that the VCF files were produced for the benchmark study only, and the output formats were under constant development.) The classes of variants and the number of variants reported in the VCF files differ significantly among the callers (Additional file 1: Figure S1A). Caller D reported only somatic variants. Caller A, B, and C reported both somatic and germline variants but the numbers vary. Our main interest is in the somatic variants that successfully pass all the filters implemented by each caller, which comprise the final set of mutations. Nonetheless, information of the somatic variants that did not pass filters can be used for comparison purposes. For example, for each caller, we screen the corresponding VCF file to check whether the mutations missed by the caller only are reported in the file, and then find the reasons for them being filtered out. Around 60% of mutations that were missed by Caller B only are not reported in the VCF file from Caller B (Additional file 1: Figure S1B), suggesting that initial requirement to be scanned by Caller B might be more stringent than others. The scale of mutation quality scores reported in the VCF varies across the callers as well (Additional file 1: Figure S2). Mutations that were detected by all callers tend to have high quality and those that were detected by a single caller tend to have low mutation quality. Nevertheless, pairwise comparison of mutation quality scores between callers shows some but not a very high level of agreement (Additional file 1: Figure S3). Furthermore, such scores from all callers are available only for a limited subset of mutations. Thus ranking all mutations detected by any caller is not feasible based on these mutation quality scores.

**READ dataset** From the mutation-calling outputs (VCFs) of the three callers (Caller H, I, and J), we extracted the final set of somatic mutations. The mutations detected by each caller are stratified by the number of callers detecting those mutations (Additional file 1: Figure S4). Caller I tends to be stringent, in that it calls a small number of mutations but most of those are shared by the other two callers. Notably, among the mutations that were detected by one or more callers, a very large fraction (median of 78%) were detected by a single caller, especially by Caller H or Caller J only. The relative numbers of mutations called by each caller varies across the 6 READ patients. Specifically, for READ-1, READ-4, and READ-5, Caller H and J detected a similar number of mutations. For READ-2 and READ-6, Caller H detected around three times as many calls as Caller J. READ-3 is unique in the sense that Caller I and J detected relatively many more mutations compared to other cases.

Since many mutations were detected by a single caller only, we explored such mutations further. First, we checked whether each mutation detected by a single caller was reported in the VCF files of other callers. Additional file 1: Figure S5 shows that it is the case for majority of the mutations. That says, those mutations were initially considered for variant detection by at least one more caller but filtered out. Unfortunately, as mentioned earlier, the full details of all filters were not available for further exploration. We also examined the coverage and the vaf of the tumor sample of such mutations. Almost all calls detected by Caller J only have very low vaf, less than 10%. The tumor vaf of the mutations detected by Caller H only covers the whole spectrum of allele fractions, and more than 80% of those are larger than 10% (data not shown).

### Analysis of validation data

**LUSC dataset** Deep-sequencing data was available for 76 genes for all LUSC patients. We used it to determine the validation status of any variant in the VCF files that are within the 76 genes. A variant is called ‘somatic’ if the tumor vaf in the deep-seq data is > 10% and the normal vaf in the deep-seq data is < 2%, otherwise, ‘non-somatic’. (This specific validation rule is determined based on our examination of the deep-sequencing data while constructing an evaluation dataset. Further details are described below). The validation results are summarized by each mutation set, defined based on the detection status by the four callers (Table 1). Notice that mutations that were detected by two or more callers tend to have a high accuracy. For example, all the 13 mutations detected by all but Caller A, or all the 6 mutations detected by Caller A and C *are* validated as ‘somatic’. Mutations that were called by a single caller tend to be enriched for false positives but some of those turned out to be false negatives for the other three callers, suggesting that a non-negligible number of sites posed difficulties in mutation-calling. For example, among the 11 mutations that were called by Caller D only, five of them are validated as ‘somatic’, i.e., Caller A, B, and C failed to detect these mutations.

**Table 1:** Validation of the mutations within the targeted regions (76 genes) of the Illumina deep-sequencing data from 16 LUSC patients

**Mutation set**	**Non-somatic**	**Somatic**
None	4292	9
Caller A only	3	4
Caller B only	1	2
Caller C only	2	0
Caller D only	6	5
Caller A and C	0	6
Caller A and D	2	3
Caller B and C	0	2
Caller B and D	0	2
Caller C and D	0	5
All but Caller D	0	3
All but Caller C	0	4
All but Caller B	3	15
All but Caller A	0	13
All callers	0	57

The variants included in any mutation output (VCF) file were divided into mutation sets based on the detection status of the four callers (rows). For each variant, validation status (columns) was determined as ‘somatic’ if the deep-sequencing data shows the signature of a somatic mutation: the vaf in the tumor sample is > 10% and the vaf in the normal sample is < 2%; otherwise, it is as ‘non-somatic’. Variants that have less than 6 reads in the original exome-seq data were discarded. One mutation was such a case.

**Figure 3:**
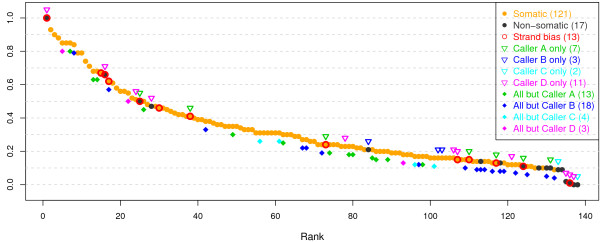
**Validation status of individual mutations within the targeted regions of the deep-sequencing data (76 genes), among those detected from 16 LUSC whole exome-seq pairs using four callers.** Mutations that were detected by at least one caller (in total, 138) are ordered by the variant allele fraction in the tumor exome-seq data. The validation status (‘somatic’ or ‘non-somatic’) was determined based on the deep-sequencing data as described in Table 1. A mutation is marked as ‘strand bias’ (red circle) when more than 95% or less than 5% of the reads carrying the variant allele are on the forward strand in the tumor exome-seq data. Mutations that were detected by a single caller only (e.g., Caller B only) or missed by a single caller only (e.g., All but Caller B) are indicated with upside down triangle or filled diamond, respectively.

The individual validation status of all the 138 mutations is shown in Figure 3. Mutations are marked as ‘Strand bias’ if more than 95% or less than 5% of the reads carrying the variant allele are on the forward strand. Out of 13 such mutations, 11 overlap with those mutations that were detected by a single caller or missed by a single caller. Not surprisingly, many of the mutations validated as ‘non-somatic’ have low tumor vaf (less than 15%). Manual examination of the five mutations validated as ‘non-somatic’ but having tumor vaf > 20% suggested that four of them are likely to be ‘germline’ mutations. Mutations in each mutation set (e.g., ‘detected by Caller A only’ or ‘detected by Caller D only’) tend to scatter around across a large range of tumor vafs. One exception is the set ‘detected by All but Caller B’, for which many of them are clustered by having a low vaf.

We further interrogated false positive and false negative sites by exploring alignment of nearby regions, distribution of base scores of the variant and the reference allele, coverage, and variant allele fraction. Some of such details for each mutation are summarized in Additional file 2. Each discrepancy has a very specific reason but broadly, there are two explanations accounting for the false positives and false negatives. One is when a mutation has low-quality information (low vaf in the tumor sample, or low coverage, or low mapping quality, or low base quality scores for the variant allele). The other is when different pre- and post-filtering criteria were applied to high or intermediate-quality mutations, i.e., mutations showing clear signs of the existence of the variant allele, but may show strand bias, repeatedly appear in the first or last few bases of the reads, etc. Nonetheless, detailed characterization couldn’t be performed, since full details of the filters were not available.

**READ dataset** Among the mutations called for the 6 READ patients, we obtained the validation status of 721 sites that were validated by the 454 technology. We first classified each mutation found in any VCF file into five validation groups. It is ‘nonMAF’ if the mutation was found in the benchmark data but did not appear in the TCGA colon working group MAF (mutation annotation format) file as of Nov 8, 2011 nor validated by the 454 technology. Otherwise, it was classified as ‘unknown’ (in the MAF file but not validated), ‘wildtype’ (no variant allele found in either the tumor or the normal sample), ‘germline’ (variant allele found in both the tumor and the normal sample), and ‘somatic’ (the variant allele is found in the tumor sample but not in the normal). We didn’t have information about how sites were chosen for validation. However, classification of the mutations by the detection status of the three callers and by the validation group suggests that only the variants initially called by Caller J were considered for validation, except for a few variants in READ-4 (Additional file 1: Table S2). It is important to be aware of such ascertainment. Otherwise, one may mistakenly compute the false positive and the false negative rates of Caller H or Caller I using the validated mutations, and may make unfair comparison with the rates of Caller J. Note that in the set of validated mutations, if a mutation is called by Caller H, then it implicitly implies that the mutation is called by both Caller H and Caller J due to the ascertainment.

Validation results for the mutations that were called by Caller J are shown in Additional file 1: Figure S6. Most of the calls detected by Caller J only were not considered for validation nor included in the MAF file (i.e., ‘nonMAF’), except for the calls from patient READ-3 and READ-4. For the two patients, numerous mutations were considered for validation and validated as wildtype. Mutations that were detected by all callers have high validation rate for most of the patients, except READ-3. For READ-3, around half of the mutations were validated as wildtype. The validation results of the mutations that were detected by Caller J and another caller also suggest somewhat unusual validation accuracy for the mutations from two patients, READ-3 and READ-4. It is plausible that the quality of samples or the experimental procedure for these patients provided a challenge to all three mutation callers.

**Figure 4:**
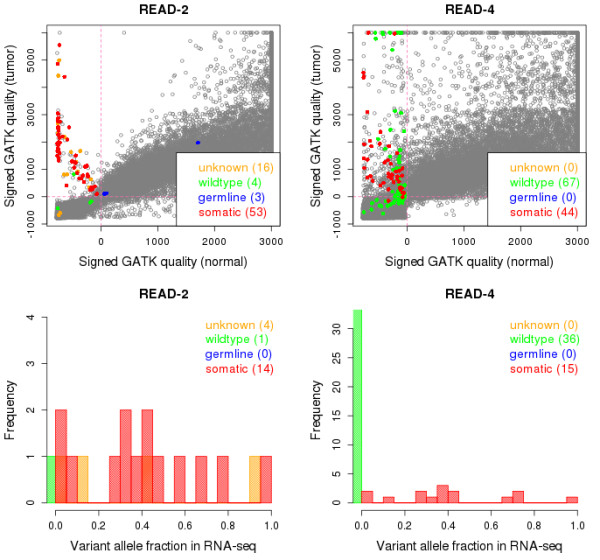
**Motivation for the pseudo- and the RNA-seq validation methods.** For two READ patients (columns), the upper row shows the *signed* GATK variant quality scores based on the tumor-normal Illumina exome-seq pairs, and the lower row shows the histogram of the variant allele fraction in the tumor Illumina RNA-seq. Note that for the READ samples, mutation calling was done by three callers (Caller H, I, and J) using SOLiD exome-seq pairs. For the upper panels, we first compiled all the variant sites reported in any VCF file based on SOLiD exome-seq pairs. Then, for each such site, using the Illumina exome-seq data, we obtained the GATK variant call quality score for each tumor (y-axis) and the normal sample (x-axis). When no variant allele was detected by the GATK UnifiedGenotyper, we flipped the sign. Mutations that were validated by the 454 sequencing technology are colored: red (somatic), blue (germline) and green (wildtype). Among the validated mutations, those with the RNA-seq depth ≥ 5x were further examined for the variant allele fraction in the tumor RNA-seq data (lower panels).

Our earlier work suggested that for Illumina sequencing data, the variant quality scores computed by the GATK UnifiedGenotyper[19] could be effectively used for visualizing overall and individual mutation qualities. Therefore, we first compiled the variants in any VCF output, which was produced based on 6 SOLiD exome-seq pairs. Then for each such variant, we obtained the *signed* GATK variant call quality scores using the corresponding Illumina exome-seq pairs (for details, see Methods). The *signed* GATK qualities are shown for two patients in Figure 4, and for all 6 patients in Additional file 1: Figure S7. We expect a somatic mutation to have a large positive *signed* GATK quality score in the tumor sample (strong support of the existence of the variant allele) and a large negative *signed* GATK quality score in the normal sample (strong support for two homozygous reference alleles). Indeed, most of mutations validated as ‘somatic’ show such characteristics. For READ-1, READ-2, READ-4 and READ-5, the overall distribution of the GATK quality scores suggests good DNA sample quality. In this case, wildtypes, germlines, and somatic mutations are well distinguished. However, for READ-3 and READ-4, the distribution of the GATK quality score seems much noisier (numerous points in the area in which biologically meaningful variants are not likely to be), suggesting poor DNA sample quality. Numerous wildtypes show expected characteristics for somatic mutations, and that is likely to be the reason for the relatively poor validation rates for these two patients in Additional file 1: Figure S6. Note that mutation-calling is done based on SOLiD sequences, but the signed GATK quality scores are computed based on the Illumina sequences. Therefore, the difficulty is not likely due to the sequencing technology but due to sample quality. We suspect that the poor quality DNA sample induces many artifactual variants causing high false positive rates, since those artifactual variants do not exist in the RNA-seq tumor sample (Figure 4). Notice that all the mutations validated as wildtypes have *zero* vaf in the RNA-seq data.

### Analysis of the deep-sequencing data for construction of an evaluation dataset

**Figure 5:**
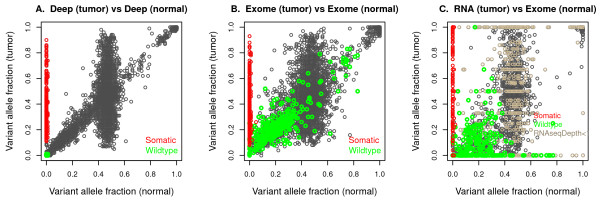
**Scatter plots of tumor vs normal variant allele fractions, using deep-seq pairs (A), exome-seq pairs (B), and tumor RNA-seq and normal exome-seq (C) from 39 LUSC patients.** Each point is a variant site detected in the tumor exome-seq data using the GATK UnifiedGenotyper. Variants detected within 76 genes (targeted regions of the deep-sequencing data) are aggregated over the 39 patients (in total, 6,692). Using the deep-sequencing data, we determined the validation status as ‘somatic’ if the tumor vaf is > 10% and the normal vaf is < 2% (334 sites; red). Among the ‘non-somatic’ ones, we further classified the variants as ‘wildtype’ if the tumor vaf is < 2% and the normal vaf is < 2% (319 sites; green).

For method development, we constructed an evaluation dataset by compiling a set of candidate somatic variants detected based on the exome-seq data and determining their validation status based on the deep-sequencing data. Our evaluation dataset consists of the 6,692 variant sites that were detected within the 76 genes using the GATK UnifiedGenotyper from 39 LUSC tumor exome-seq data (for further details, see Methods). To determine the validation status of these sites, we first examined the distribution of variant allele fractions in the deep-seq tumor-normal pairs (Figure 5A). The scatter plot shows good separation between somatic mutations from other types of variants. A somatic mutation is characterized by having a variant allele in the tumor sample but not in the normal sample. On the left boundary of the plot, there are many points for which the tumor vaf is reasonably large (or away from zero, e.g., > 5%) but the normal vaf is almost zero (e.g., < 2%). These points are well separated from the other points, especially when the normal vaf is larger than zero (e.g., > 2%). The distribution of the vafs in the normal samples clearly has three modes (near zero, 45%, and 100%), corresponding to genotypes with zero, one, and two variant alleles. Presumably, the alignment bias preferring the reference allele resulted in the centre mode around 45% rather than 50%. When the normal vaf is near the centre mode (i.e., the normal is heterozygous), the tumor vafs are vertically spread around, suggesting that varying degrees of normal contamination exist in the tumor samples and the loss of heterozygosity regions. Interestingly, there are numerous variants for which the normal vaf departs from the three modes but the correspondence between the tumor vaf and the normal vaf is very high. In particular, most of these variants have the normal vaf less than half. Since these variants have both the tumor and normal vafs in the range suggesting for the existence of a variant allele, we name these as ‘germline-like’ variants. Alignment biases are not likely to explain numerous occurrence of these ‘germline-like’ variants fully. We suspect that a large fraction of these variants appeared due to artifactual variant alleles that were caused by sequencing technology, since a substantial number of the variants exhibit extreme levels of strand bias (Additional file 1: Figure S8). Specifically, among the 787 germline-like variants for which the normal vaf is between 5% and 35%, around 50% variants exhibit very strong strand bias in the tumor sequence data (more than 95% or less than 5% of reads carrying the variant allele are on the forward strand). In contrast, among the 2,716 variants for which the normal vaf is in a more expected range (40% – 60%) for heterozygous genotype for the normal sample, only 1.5% exhibit such extreme strand bias. Below, we further discuss that base mis-calling can cause numerous artifactual variant alleles that are characterized by extreme strand bias, in contrast to the reference alleles.

We also examined the GATK variant quality scores (Additional file 1: Figure S9A). Such scores indirectly combine the vaf with sequence depth for each sample. Since we flipped the sign when no alternative allele is found by the GATK, the points on the left boundary with positive values for the tumor sample and negative values for the normal sample are strongly supported to be somatic mutations. Those points are reasonably well separated from other points in the diagonal. After further examination of the distribution of the vafs as well as the signed GATK quality scores, we determined the validation status for each of the 6,692 sites based on the deep-seq pairs (gold-standard data). A variant is called ‘somatic’ (334 sites; ∼ 5%) if the tumor vaf is > 10% and the normal vaf is < 2%, otherwise, ‘non-somatic’. An alternative criterion for a ‘somatic’ mutation is that the signed GATK quality scores for the tumor sample and for the normal sample are > 200 and < -100, respectively. The two criteria mismatch only for 17 sites. Rather than making subjective judgement for these sites, we have exclude these 17 ambiguous sites from our final evaluation set.

### Reasons for artifactual variants

In the evaluation dataset, we validated the variants found in the exome-seq data using the deep-sequencing data. Among the variants validated as ‘non-somatic’, there are numerous cases for which both the tumor and the normal vafs are very low (<2%) in the deep-sequencing data. We classified these as ‘wildtypes’ (319 sites) and visualized for comparison with somatic ones and investigated the reasons for mis-calling. Notice that many of these wildtypes look like germline variants in the original exome-seq data (Figure 5B and Additional file 1: Figure S9B). These artifactual variants also exist in the tumor RNA-seq data (Figure 5C). Our exploration reveals a remarkable difference in the strand bias pattern between the somatic mutations and the wildetypes in the tumor samples (Additional file 1: Figure S10). (Strand bias is computed as the fraction of reads on the forward strand among the reads carrying the allele of interest.) For somatic mutations, the fraction of reads on the forward strand is very similar between the variant allele and the reference allele. For wildtypes, the strand bias of the variant allele is extreme in that almost all the reads carrying the variant allele are either on the forward strand or the reverse strand. Although weaker than the tumor exome-seq, the RNA-seq data exhibits a similar behavior. Thus, the sources causing these wildtypes are not the characteristics of the protocol used for exome-sequencing or RNA-sequencing, but rather are likely to be consequences of the Illumina sequencing technology. Even for the deep-sequencing data, numerous false variants seem to occur due to strand bias. The ‘germline-like’ variants based on the deep-seq pairs show a very different strand bias pattern compared to the somatic ones (Additional file 1: Figure S8). To a large extent, we believe that such false variants occur due to base-calling errors in the raw sequencing reads [24]. Some sequence contexts are more prone to base-calling errors than others, and these errors may occur in only one direction [17,18]. Indeed, the direction of the strand bias is the same across the different sequenced samples such as tumor exome-seq and the normal exome-seq (data not shown).

### Pseudo- and RNA-seq validation methods

**Pseudo-validation method** In the absence of a gold standard data, direct comparison among calls from different mutation callers is difficult. Each caller reports information for a subset of genomic positions, and these sets do not entirely overlap. To overcome this difficulty, i.e., to provide reasonable and consistent measure of mutation quality across all positions that are reported by any mutation caller, we used a publicly available variant caller, GATK UnifiedGenotyper, to build our own pseudo-caller. Specifically, for each variant of interest, we computed the *signed* GATK variant quality score for each tumor and normal sample (for details, see Methods). With a given threshold *q*_*t*_ for the tumor sample, and *q*_*n*_ for the normal sample, we define pseudo-positives as variants for which the signed GATK quality from the tumor is >*q*_*t*_ and the signed GATK from the normal is <*q*_*n*_. Note that the pseudo-caller is for comparison purposes only, and is not to be treated as an independent mutation caller.

**RNA-seq validation method** RNA-sequencing is often done for tumor samples, since the expression pattern of the tumor sample is of great interest in many cancer projects. If such tumor RNA-seq data is available as well as the original exome-seq pairs used for mutation calling, it can be used as partial validation data. Due to the variation in the expression levels across the genes, RNA-seq generally shows a significantly larger variation than DNA-seq data in the number of reads carrying the variant allele.

To construct a validation dataset using the RNA-seq data for the tumor sample, we examined the variant allele fraction (vaf) in the RNA-seq data together with the vaf in the normal exome-seq data. We restricted our analysis to those variants that have RNA-seq depth >10x. (In our datasets, 30-40% of sites satisfy this restriction.) Then, with a given threshold for tumor and the normal, *f*_*t*_ and *f*_*n*_, respectively, we define positives as such variants for which the tumor RNA-seq vaf exceeds *f*_*t*_ and the normal exome-seq vaf is less than *f*_*n*_.

### Evaluation of pseudo- and RNA-seq validation methods

To assess the performance of the pseudo-validation method, we ranked the variants in the evaluation set using the GATK quality score obtained from the tumor exome-seq, given that the signed GATK quality score from the normal sample is less than -50. We mostly used only the tumor sample for ranking, since most of difficulties in calling somatic mutations seem to occur due to heterogeneous aspects of the tumor sample. For the normal sample, it suffices to set a threshold to exclude any sites with a variant in the germline. We also attempted to filter out artifactual variant alleles by introducing a criterion based on the strand bias pattern in the tumor exome-seqs. If the variant allele shows much more extreme strand bias compared to the reference allele, specifically, more than 95% or less than 5% of the variant alleles are on the forward strand, while less than 70% or larger than 30% of the reference alleles are on the forward strand, then we filtered out the mutation sites.

The performance of the RNA-seq validation method was assessed using the 1,945 variants (∼30% of the total 6,692 sites) in the evaluation dataset that have the RNA-seq depth ≥ 10x. We used the tumor vaf to rank the variants, given that the normal vaf in the exome-seq is less than 2%. To filter out artifactual variant alleles, we filtered out a mutation site if both the tumor exome-seq and the tumor RNA-seq showed strong strand bias (more than 95% or less than 5% of the reads are on the forward strand) for the variant allele. The reason that we applied different filtering criterion is because for the RNA-seq data, the correspondence of the strand bias between the variant allele and the reference allele is much weaker even for the “true” variants (Additional file 1: Figure S10C) but existing exome-seq data can help for filtering out artifactual variants.

**Figure 6:**
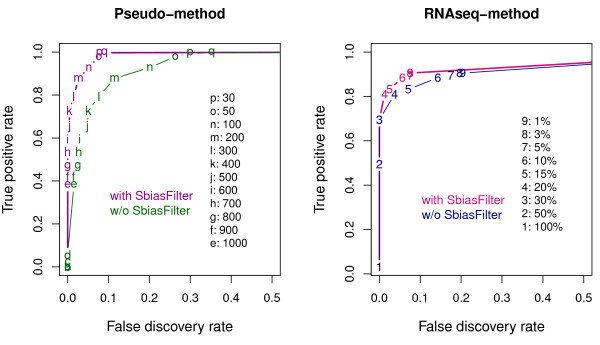
**ROC-like curves summarizing the performances of the pseudo- and the RNA-seq validation methods.** For each method, across the range of thresholds for the tumor sample, we computed the true positive rate (y-axis) at each false discovery rate (x-axis). The evaluation was done based on the 6,692 variant sites detected within 76 genes from 39 LUSC exome-seq pairs, for which, 334 sites were validated as ‘somatic’ based on the deep-sequencing data. For the pseudo-validation method, across the range of thresholds for the tumor GATK quality scores (marked with letters), a site was identified as pseudo-positive if the tumor score is larger than the threshold and the signed GATK quality score for the normal sample is less than -50. When ‘SbiasFilter’ is applied, a site becomes non-somatic if more than 95% or less than 5% of the variant alleles but less than 70% or larger than 30% of the reference alleles are on the forward strand. For the RNA-seq validation method, across the range of thresholds for the tumor RNA-seq vaf, a site was identified as positive if the tumor RNA-seq vaf is larger than the threshold and the normal exome-seq vaf is less than 2%. When ‘SbiasFilter’ is applied, a site becomes non-somatic if more than 95% or less than 5% are on the forward strand for the variant allele in both the tumor exom-seq and the RNA-seq data.

The performances of the two methods were summarized by ROC-like curves, showing the true positive rate at each false discovery rate (Figure 6). (We used the false discovery rate (FDR) instead of the false positive rate since the evaluation dataset consists of the ascertained variants obtained using the GATK UnifiedGenotyper. Thus, only relative false positive rates are meaningful. Instead, we chose to use FDR here since it has very intuitive scale and it won’t change the conclusion. Note that we examined the full spectrum of stringency levels by varying cut-offs for the GATK quality scores and for the tumor vaf in the RNA-seq data.) As expected, both validation approaches show improved performances with the strand bias filters. For the pseudo-validation method, for example, the signed GATK quality score in the tumor sample exceeds 300 for 287 sites. Among these, 265 sites are validated as ‘true’ somatic mutations (based on the gold-standard deep-sequencing data) detecting 80% of the 334 somatic mutations. The remaining 22 sites are false positives, but with the strand bias filter, 18 of them are removed, dropping the false discovery rate from 8% to 1.5%. For the RNA-seq validation method, the vaf in the RNA-seq is larger than 10% for 98 sites. Among these, 84 sites are validated as ‘true’ somatic mutations detecting 88% of the 95 somatic mutations with the RNA-seq depth > 10x. The remaining 14 sites are false positives. Nine of those were discarded using the strand bias filter, dropping the false discovery rate from 15% to 5.6%. Note that we conceived the strand bias filter based on the insights learned from the strand bias pattern in the exome-seq and the RNA-seq data in the evaluation dataset. Therefore, re-applying the strand bias filter to the same data will obviously improve the performances. Nonetheless, we keep this strand bias filter for the evaluation of the two validation methods using the benchmark data, since a similar strand bias pattern is likely to be observed using only the data from the additional 23 LUSC patients that were not used for the benchmark study. Notice that for the RNA-seq validation method, the true positive rate is only 90% even with the 1% threshold for the RNA-seq vaf. It is because some of the true variants that existed in the DNA exome-seq data did not appear in the RNA-seq due to the variation in the expression level as well as the sequencing depth.

### Comparing the performances of callers in the benchmark data using the pseudo- and RNA-seq validation methods, and latent class models

We applied the pseudo- and the RNA-seq validation methods to the whole exome benchmark data from 16 LUSC patients. For each validation method, we picked a specific cut-off for convenience and illustration purposes, but across a range of reasonable cut-offs, the qualitative conclusions were similar. For the pseudo-method, we validated mutations as somatic if the signed GATK quality scores for the tumor sample and for the normal sample are > 200 and <-50, respectively. For the RNA-seq method, for the mutations with the RNA-seq depth ≥10x (∼ 38% out of the 5,380 mutations in the exome benchmark data; Additional file 1: Figure S11), we validated them as somatic if the vaf in the RNA-seq >10% and the vaf in the normal exome-seq <2%. The chosen cut-offs for each validation method roughly correspond to a false discovery rate less than 3% and the true positive rate larger than 80% based on the analyses of the evaluation dataset constructed using data from 39 LUSC patients. However, the absolute values should not be emphasized since the features of the whole exome benchmark data are likely different from the sites within the 76 genes for which the evaluation dataset was constructed. For the two validation methods, we attempted to remove the artifactual variants by applying the strand bias filters.

When submitted to the pseudo-validation method, around 75% of the mutations called by a single caller only (1,231 sites) were validated as false positives. Notice that majority of the mutations in the upper row in Figure 2 have disappeared from Additional file 1: Figure S12, while around 85% of the mutations that were detected by three callers (1,491) have been retained. When submitted to the RNA-seq validation method, a similar observation is made (Additional file 1: Figure S13). Nonetheless, among the 1,959 sites that were called by one or more callers and validated by both the pseudo- and the RNA-seq methods, 553 sites (∼ 27%) have different validation status, implying that validation can be quite challenging. A part of these differences (104 sites) occurred for the sites that were detected by all callers.

For the mutations within the targeted regions of deep-sequencing data (76 genes), validation status based on a gold-standard data is available. Thus, for those mutations, we compared the gold-standard (GS) validation status with the status based on the pseudo- or the RNA-seq validation method. Out of 138 sites, 14 sites are validated as somatic by the GS method but not by the pseudo method, and vice versa for 3 sites. Almost all of the 14 former sites have low vaf (10% – 15%) in tumor exome-sequence data, and that may explain why missed by the pseudo method. Out of 51 mutations where RNA-seq validation can be applied (depth > 10x), 6 sites are validated as somatic by the GS method but not by the RNA-seq method, and vice versa for 3 sites. The former 6 sites tend to have low-to-medium RNA-seq coverage, mostly less than 30x, and this might be the reason for losing the true variant allele. Two out of the latter 3 sites have very low vaf (<15%) for both tumor deep-seq and the exome-seq data. Finally, when the pseudo-validation status was compared with the RNA-seq validation status, 10 out of 51 mutations (∼ 20%) show different status. It is difficult to know what are the genuinely correct status for all these discrepancies, but these observations illustrate the challenges in mutation-calling.

We also employed latent class models, which have been repeatedly used to compare multiple diagnostic tests in medical studies when there is no gold standard data [20,21,25-28]. In the benchmark study, multiple mutation callers were applied to the same tumor-normal paired sequence alignments, and the decision was made on whether each position in the alignments is a somatic mutation or not. In the absence of gold standard validation data, latent class models offer a convenient statistical framework within which the false positive and the false negative rates are estimated by treating the true mutation status as a latent variable (for more details, see Methods). We fitted the latent class model using the set of mutations within the 76 genes, and the set consisting the whole exome data. For the set using the 76 genes, the model assuming conditional independence among the callers fits the data pretty well (Pearson chi-square goodness of fit statistic is 11 with degrees of freedom of 6). For the whole exome data, the conditional independence model does not fit the data as well, but the latent class model with random effects having an additional parameter improves the fit significantly (Pearson chi-square goodness of fit statistic drops from 233 to 121, and the fitted counts look more sensible). Note that our model is equivalent to the 2LCR1 model in Qu et al. [20], except that we let the variance component be shared between ‘somatic’ and ‘non-somatic’ sites. We decided to use this model for the exome data, since more general models do not improve the fit much nor change qualitative conclusions. The observed and the fitted counts using the latent class models are summarized in Additional file 1: Table S3.

**Figure 7:**
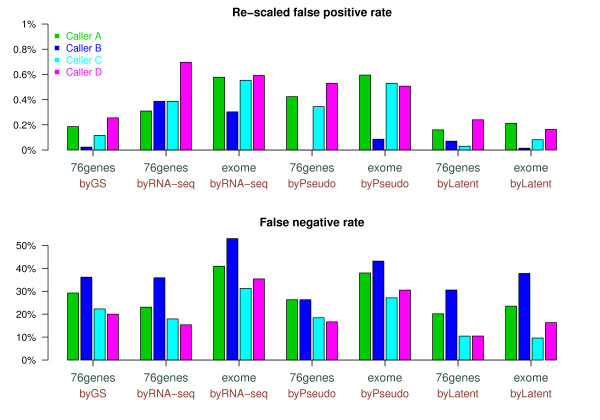
**Estimated (re-scaled) false positive rate (upper) and the false negative rates (lower), using the mutations across whole exome (‘exome’) or within the 76 genes (‘76genes’) in the LUSC benchmark data.** We applied four approaches: validation using the gold-standard deep-sequencing data (‘byGS’), the RNA-seq validation method (‘byRNA-seq’), the pseudo-validation method (‘byPseudo’), and the latent class models (‘byLatent’).

In Figure 7, we summarized the false positive (FP) and the false negative (FN) rates estimated using two datasets (76 genes or whole exome) by four validation methods: gold-standard method using the deep-sequencing data, pseudo-method using the GATK quality scores, RNA-seq validation method utilizing the variant allele fraction in RNA-seq, and the latent-class models. Note that the gold-standard validation is not applicable to the whole exome-data. For the pseudo- and the RNA-seq methods, the thresholds controlling the performance were picked by us for convenience. The latent class models directly estimate the false positive and false negative rates based on a statistical model. Since absolute values of FP rates are less emphasized for our dataset, for an easier comparison, we re-scaled the FP rates across the four validation methods. Within the 76 genes, around 3% of the all variants compiled from VCF files were validated as ‘somatic’ by the GS method. We assume that the same portion of the evaluated sites are ‘somatic’ in other datasets, and re-scaled the FP rates accordingly.

Overall, relative performances of the four callers were similar across the different datasets and methods. Caller B is stringent and other three callers show few distinctive characteristics. There is one exception. When the mutations within the 76 genes are validated by the RNA-seq method, Caller B shows an unusually high false positive rate. Our manual examination suggests that the unusually high false positive rate of the Caller B is likely due to the few sites for which the RNA-seq missed the variant allele presented in low fraction (<20%) in both the deep-seq and the exome-seq data. Therefore, one needs to be cautious when utilizing RNA-seq for validation of a small dataset. Often, Caller C performs slightly better than Caller A and D by having a lower false positive rate as well as a lower false negative rate, i.e., located in a lower left part compared to Caller A and D in Additional file 1: Figure S14. In particular, the latent class models clearly prefer Caller C, implying that high fraction of the calls detected by Caller C is shared with other callers. However, systematic comparison of the performances was not feasible, since we were not able to explore the full spectrum of the performances of each caller due to limited information.

## Discussion

We observed a number of sites exhibiting artifactual variant alleles while we were constructing the evaluation dataset using the deep-sequencing and the RNA-seq data in addition to the original exome-seq data. Most of the artifactual variants show an extreme level of strand bias for the variant allele, regardless of the corresponding level in the reference allele. Such artifactual variants are present not only in the tumor sample but also in the normal sample. In particular, on many occasions, the artifactual variants occur simultaneously in both tumor and normal samples, creating ‘germline-like’ variants, based on the exome-seq data. Moreover, a substantial fraction of such variants are also present in the tumor RNA-seq data. The accumulation of such errors repeatedly at the same genome location across different biological samples suggests that those artifactual variants are likely to occur due to the errors in the sequencing technologies, cf. Meacham et al. [17]. The ‘germline-like’ variants based on the deep-sequencing data also exhibit a strand bias pattern similar to the artifactual variants found in the exome-seq data.

For somatic mutation-calling, the joint occurrence of the artifactual variants in both tumor and normal samples could be useful for filtering out ‘non-somatic’ mutations. Any position exhibiting the presence of a variant allele in the normal sample will be discarded, and thus the artifactual variant in the tumor sample when the normal sample also carries it. For a similar reason, many of artifactual variants in the tumor RNA-seq data were filtered out by our RNA-seq validation method. Interestingly, substituting the normal exome-seq data by the normal deep-seq data hurts the performance of the RNA validation method. The deep-sequencing data seems to have a higher quality and thus contains many fewer artifactual variants, and thus filtering out artifactual variants in the tumor RNA-seq data was less effective. A better characterization of artifactual variants remains as our future work.

In this study, we introduced two validation approaches, the pseudo- and the RNA-seq validation methods. The two approaches, however, should not be considered as alternative mutation-calling methods. Our validation methods were developed to overcome the challenge of comparison, rather than for correcting all the biases or errors affecting mutation-calling. In particular, the performance of the pseudo-validation method depends on how effectively the GATK UnifiedGenotyper calls a variant in the tumor and the normal samples. Even though we observed that on many occasions it proved its usefulness in somatic-mutation analyses, GATK was developed for normal samples not for tumor samples. Furthermore, some of the mutation-callers used for the benchmark study may share with the GATK the underlying statistical models or methods, which could result in biased inferences. However, our small exercise in replacing GATK UnifiedGenotyper with Samtools mpileup suggests this was not the case.

## Conclusions

TCGA conducted benchmark studies comparing multiple mutation-callers on the same sequence pairs (BAMs). Our work attempted to characterize the discrepancies among the callers in two benchmark datasets, and provide guidelines for the analysis of such comparative data. To assess the performances of mutation callers, we have introduced four approaches estimating the sensitivity and the specificity of each caller.

Our analyses revealed that the discrepancies among the callers not only occur at sites with low-information (low sequence depth, low mapping quality, or low variant allele fraction), but also at sites with intermediate or high-quality information. Specifically, a large fraction of calls missed by a single caller in the LUSC benchmark dataset exhibit strong evidence for the existence of a variant allele. Investigation of raw mutation-outputs (VCF) suggested that the uniquely missing caller initially included such calls as candidate somatic mutations but implemented a filter removing those. The details of these filters were not available in the VCF files for this benchmark study, but this recognition emphasizes the value of full details for a comprehensive analysis. In addition to that, a more comprehensive analysis can be performed if somatic mutation qualities are included in the VCF files, allowing ranking of mutations within each caller and thus varying the stringency level of the caller by changing the false positive rate. Then the full ROC curve showing the performance of each caller can be examined.

Strand bias is one of well-known features producing artifactual variants in high-throughput sequencing data, and we presume at least some of the callers used for the benchmark study have implemented filters for strand bias. Nevertheless, our analysis of the validation data for the LUSC samples suggests that the specifications of such filters are likely to vary considerably across the callers. When we marked the mutations that have an extreme level of strand bias for the variant allele in the tumor exome-seq sample, most of them accounted for the discrepancies.

Our work calls for extra caution in comparing the performances of multiple callers based on an *ascertained* validation data, since ‘not attempted to be validated’ mutations should be distinguished from ‘attempted to be validated’ mutations. For the READ dataset, validation information for 721 sites was available, but not the information about the ascertainment, i.e., how those sites were chosen for validation. By classifying the mutations based on the detection status of the three callers and the validation status, we learned that almost all of those 721 sites were initially called by one particular caller. Presumably, the experimental validation of those sites was performed for evaluation of that particular caller’s performance, and not for comparison of multiple callers. Such validation information can be used for learning about individual errors of other callers but should not be used for evaluating the performance of other callers.

Somatic mutation-calling based on the high-throughput sequencing data is a rapidly evolving field. Currently, a limited number of somatic mutation-callers are publicly available [29-32], but many more are likely to appear in the near future. Our paper focused on understanding the discrepancies and highlighting the challenges in comparing multiple callers. With more details in the mutation outputs, such as mutation quality scores and the details of filters, another interesting question to address is how to combine the calls across multiple callers. A recent study by Lower et al. [33] tackled a similar problem by assigning an FDR confidence score for each call from multiple callers, but their method requires replicate sequencing of at least one of the tumor or the normal sample. When only a pair of tumor-normal sequence data are available for mutation-calling, combining the calls incorporating the information in the outputs from multiple callers is another upcoming problem.

## Methods

### Obtaining *signed* GATK variant quality scores

The GATK UnifiedGenotyper[19] is one of publicly available variant callers. For a sample of interest, it takes the sequence data (BAM) and detects SNPs by default. The outputs are summarized in a VCF file, which includes a list of variant positions along with the associated call quality scores (‘QUAL’ column in the VCF). The software can produce calls only at variant sites but also at any callable site regardless of confidence. We used an option for the latter case to obtain variant call quality for all positions of interest. An example of run command is as follow.

java -jar GenomeAnalysisTK.jar -R human_reference.fasta -T UnifiedGenotyper -I input.bam –computeSLOD -L positions_of_interest.bed –output_mode EMIT_ALL_SITES -o output.vcf

For a given list of genomic positions, we ran the GATK UnifiedGenotyper (version 1.5-3) on each tumor sample and its matched normal sample. We forced it to emit variant call quality for all positions. When no variant allele was found, the variant call quality represents a confidence level for the homozygous reference genotype. To differentiate such positions, we flipped the sign of the variant call quality. Therefore, a large negative value is a strong support for the non-existence of a variant allele, and a large positive value is a strong support for the existence of a variant allele.

### Constructing an evaluation dataset

For method development, we aimed to construct an evaluation dataset that consists of a set of candidate somatic variants for which the validation status is assumed to be known (i.e., determined based on the gold-standard data). For LUSC patients, variants called based on the exome-sequence data can be validated based on the deep-sequencing data for 76 genes. We constructed an evaluation set using the sequence data from 39 LUSC patients, which include an additional 23 patients. Note that the exome-seq pairs, the tumor RNA-seq, and the deep-sequence pairs were available for a much larger set of LUSC patients than the 16 patients used for the benchmark mutation-calling comparison.

To build an evaluation set that includes all somatic mutations, we first detected variants in each tumor exome-seq data using the GATK UnifiedGenotyper[19] with a very lenient stringency level (GATK variant quality score ≥ 5):

java -jar GenomeAnalysisTK.jar -R human_reference.fasta -T UnifiedGenotyper -I input.bam –computeSLOD -L exome_annotations.bed –output_mode EMIT_VARIANTS_ONLY -stand_call_conf 5 -o output.vcf

When cross-checked with the mutation-call data available for the 16 patients, all but three of the 5,380 mutations in the benchmark data were detected. From the 39 LUSC patients, 8,828 variants were detected within the 76 genes (the targeted regions of deep-sequence data). Since we aimed to build a gold standard validation set with a high accuracy, we retained only the variants that have high tumor deep-seq depth with ≥ 100x (8,033). By requiring the tumor and the normal exome-seq depth to be ≥ 10x, and also by removing 17 ambiguous variants through manual examination, we obtained 6,692 variants as our final set. After examining the deep-sequencing data at these variants (see Results), we determined the validation status. A variant is called ‘somatic’ (334 sites; ∼ 5%) if the tumor deep-seq vaf is > 10% and the normal deep-seq vaf is < 2%, otherwise, ‘non-somatic’. An alternative criterion for a ‘somatic’ mutation is that the signed GATK quality scores for the tumor sample and for the normal sample are > 200 and < -100, respectively.

### Utilizing latent class models

Here we provide a quick review on the latent class model assuming that mutation callers were developed based on independent algorithms.

Suppose that *K* mutation-callers evaluate *N* positions for their mutation status (1=somatic, 0=non-somatic). Let *Y*_*i*,*k*_ denote the observed outcome of the *i*th position by the *k*th caller, and *Y*_*i*_=(*Y*_*i*,1_,…,*Y*_*i*,*K*_) be the vector of outcomes over all callers for the *i*th position. Let *D*_*i*_ denote the true mutation status of the *i*th position, and *η* the mutation prevalence, *P*(*D*_*i*_=1), assumed independent of *i*. Then, the probability of observing *Y*_*i*_ is computed as follows:

$$\;P(Y_{i})=(1-\eta )P(Y_{i}|D_{i}=0)+\mathrm{\eta P}(Y_{i}|D_{i}=1)$$

Assuming that callers behave conditionally independently given the true mutation status,

$$\;P(Y_{i}|D_{i}\;=\;0)\;=\;P(Y_{i,1},\ldots ,Y_{i,K}|D_{i}\;=\;0)\;=\;\overset{K}{\underset{k=1}{\prod }}P(Y_{i,k}|D_{i}=0)$$

$$\;P(Y_{i}|D_{i}\;=\;1)\;=\;P(Y_{i,1},\ldots ,Y_{i,K}|D_{i}\;=\;1)\;=\;\overset{K}{\underset{k=1}{\prod }}P(Y_{i,k}|D_{i}=1)$$

Note that *P*(*Y*_*i*,*k*_=1|*D*_*i*_=0) and *P*(*Y*_*i*,*k*_=0|*D*_*i*_=1) are the false positive and the false negative rates of the *k*th caller, respectively. Therefore, there are 1+2*K* parameters: the mutation prevalence *η*, and the false positive and the false negative rates of each of the *K* mutation-callers.

## Abbreviations

LUSC: Lung squamous cell carcinoma; READ: Rectum adenocarcinoma; BAM: Binary version of sequence alignment/map (SAM) format; VCF: Variant calling format; MAF: Mutation annotation format.

## Competing interests

The authors declare that they have no competing interests.

## Authors’ contributions

SYK participated in the design of the study, carried out statistical analyses and drafted the manuscript. TPS conceived the study, participated in its design and helped to draft the manuscript. Both authors read and approved the final manuscript.

## Supplementary Material

Additional file 1 Supplementary tables and figures. Click here for file

Additional file 2 **Details of the individual mutations that were detected by a single caller only or missed by a single caller only, among those detected within the 76 genes from 16 LUSC exome-seq pairs.** The details include the validation status, detection status based on the four callers, and the *signed* GATK quality score, sequencing depth, variant allele fraction, measurement of strand bias in the deep-seq, exome-seq and RNA-seq data. Click here for file

## References

[B1] Wong KM Hudson TJ McPherson JD Unraveling the genetics of cancer: genome sequencing and beyond Annu Rev Genomics Hum Genet 2011 12 407 430 10.1146/annurev-genom-082509-141532 21639794

[B2] Meyerson M Gabriel S Getz G Advances in understanding cancer genomes through second-generation sequencing Nat Rev Genet 2010 11 10 685 696 10.1038/nrg2841 20847746

[B3] Stratton MR Exploring the genomes of cancer cells: progress and promise Science 2011 331 6024 1553 1558 10.1126/science.1204040 21436442

[B4] Ding L Wendl MC Koboldt DC Mardis ER Analysis of next-generation genomic data in cancer: accomplishments and challenges Hum Mol Genet 2010 19 R2 R188 196 10.1093/hmg/ddq391 20843826 PMC2953747

[B5] Le Gallo M O’Hara AJ Rudd ML Urick ME Hansen NF O’Neil NJ Price JC Zhang S England BM Godwin AK Sgroi DC Hieter P Mullikin JC Merino MJ Bell DW NIH Intramural Sequencing Center (NISC) Comparative Sequencing Program Exome sequencing of serous endometrial tumors identifies recurrent somatic mutations in chromatin-remodeling and ubiquitin ligase complex genes Nat Genet 2012 44 12 1310 1315 10.1038/ng.2455 23104009 PMC3515204

[B6] Zang ZJ Cutcutache I Poon SL Zhang SL McPherson JR Tao J Rajasegaran V Heng HL Deng N Gan A Lim KH Ong CK Huang D Chin SY Tan IB Ng CCY Yu W Wu Y Lee M Wu J Poh D Wan WK Rha SY So J Salto-Tellez M Yeoh KG Wong WK Zhu YJ Futreal PA Pang B Exome sequencing of gastric adenocarcinoma identifies recurrent somatic mutations in cell adhesion and chromatin remodeling genes Nat Genet 2012 44 5 570 574 10.1038/ng.2246 22484628

[B7] Puente XS Pinyol M Quesada V Conde L Ordóñez GR Villamor N Escaramis G Jares P Beà S González-Díaz M Bassaganyas L Baumann T Juan M López-Guerra M Colomer D Tubío JMC López C Navarro A Tornador C Aymerich M Rozman M Hernández JM Puente DA Freije JMP Velasco G Gutiérrez-Fernández A Costa D Carrió A Guijarro S Enjuanes A Whole-genome sequencing identifies recurrent mutations in chronic lymphocytic leukaemia Nature 2011 475 7354 101 105 10.1038/nature10113 21642962 PMC3322590

[B8] Varela I Tarpey P Raine K Huang D Ong CK Stephens P Davies H Jones D Lin ML Teague J Bignell G Butler A Cho J Dalgliesh GL Galappaththige D Greenman C Hardy C Jia M Latimer C Lau KW Marshall J McLaren S Menzies A Mudie L Stebbings L Largaespada DA Wessels LFA Richard S Kahnoski RJ Anema J Exome sequencing identifies frequent mutation of the SWI/SNF complex gene PBRM1 in renal carcinoma Nature 2011 469 7331 539 542 10.1038/nature09639 21248752 PMC3030920

[B9] Biankin AV Waddell N Kassahn KS Gingras MC Muthuswamy LB Johns AL Miller DK Wilson PJ Patch AM Wu J Chang DK Cowley MJ Gardiner BB Song S Harliwong I Idrisoglu S Nourse C Nourbakhsh E Manning S Wani S Gongora M Pajic M Scarlett CJ Gill AJ Pinho AV Rooman I Anderson M Holmes O Leonard C Taylor D Pancreatic cancer genomes reveal aberrations in axon guidance pathway genes Nature 2012 491 7424 399 405 10.1038/nature11547 23103869 PMC3530898

[B10] Ding L Ley TJ Larson DE Miller CA Koboldt DC Welch JS Ritchey JK Young MA Lamprecht T McLellan MD McMichael JF Wallis JW Lu C Shen D Harris CC Dooling DJ Fulton RS Fulton LL Chen K Schmidt H Kalicki-Veizer J Magrini VJ Cook L McGrath SD Vickery TL Wendl MC Heath S Watson MA Link DC Tomasson MH Clonal evolution in relapsed acute myeloid leukaemia revealed by whole-genome sequencing Nature 2012 481 7382 506 510 10.1038/nature10738 22237025 PMC3267864

[B11] Shah SP Roth A Goya R Oloumi A Ha G Zhao Y Turashvili G Ding J Tse K Haffari G Bashashati A Prentice LM Khattra J Burleigh A Yap D Bernard V McPherson A Shumansky K Crisan A Giuliany R Heravi-Moussavi A Rosner J Lai D Birol I Varhol R Tam A Dhalla N Zeng T Ma K Chan SK The clonal and mutational evolution spectrum of primary triple-negative breast cancers Nature 2012 486 7403 395 399 22495314 10.1038/nature10933 PMC3863681

[B12] Chapman MA Lawrence MS Keats JJ Cibulskis K Sougnez C Schinzel AC Harview CL Brunet JP Ahmann GJ Adli M Anderson KC Ardlie KG Auclair D Baker A Bergsagel PL Bernstein BE Drier Y Fonseca R Gabriel SB Hofmeister CC Jagannath S Jakubowiak AJ Krishnan A Levy J Liefeld T Lonial S Mahan S Mfuko B Monti S Perkins LM Initial genome sequencing and analysis of multiple myeloma Nature 2011 471 7339 467 472 10.1038/nature09837 21430775 PMC3560292

[B13] Stransky N Egloff AM Tward AD Kostic AD Cibulskis K Sivachenko A Kryukov GV Lawrence M Sougnez C McKenna A Shefler E Ramos AH Stojanov P Carter SL Voet D Cortés ML Auclair D Berger MF Saksena G Guiducci C Onofrio R Parkin M Romkes M Weissfeld JL Seethala RR Wang L Rangel-Escareño C Fernandez-Lopez JC Hidalgo-Miranda A Melendez-Zajgla J The mutational landscape of head and neck squamous cell carcinoma Science 2011 333 6046 1157 1160 10.1126/science.1208130 21798893 PMC3415217

[B14] Lee W Jiang Z Liu J Haverty PM Guan Y Stinson J Yue P Zhang Y Pant KP Bhatt D Ha C Johnson S Kennemer MI Mohan S Nazarenko I Watanabe C Sparks AB Shames DS Gentleman R de Sauvage FJ Stern H Pandita A Ballinger DG Drmanac R Modrusan Z Seshagiri S Zhang Z The mutation spectrum revealed by paired genome sequences from a lung cancer patient Nature 2010 465 7297 473 477 10.1038/nature09004 20505728

[B15] Forster M Forster P Elsharawy A Hemmrich G Kreck B Wittig M Thomsen I Stade B Barann M Ellinghaus D Petersen BS May S Melum E Schilhabel MB Keller A Schreiber S Rosenstiel P Franke A From next-generation sequencing alignments to accurate comparison and validation of single-nucleotide variants: the pibase software Nucleic Acids Res 2013 41 e16 10.1093/nar/gks836 22965131 PMC3592472

[B16] Nielsen R Paul JS Albrechtsen A Song YS Genotype and SNP calling from next-generation sequencing data Nat Rev Genet 2011 12 6 443 451 10.1038/nrg2986 21587300 PMC3593722

[B17] Meacham F Boffelli D Dhahbi J Martin DI Singer M Pachter L Identification and correction of systematic error in high-throughput sequence data BMC Bioinformatics 2011 12 451 10.1186/1471-2105-12-451 22099972 PMC3295828

[B18] Nakamura K Oshima T Morimoto T Ikeda S Yoshikawa H Shiwa Y Ishikawa S Linak MC Hirai A Takahashi H Altaf-Ul-Amin M Ogasawara N Kanaya S Sequence-specific error profile of Illumina sequencers Nucleic Acids Res 2011 39 13 e90 10.1093/nar/gkr344 21576222 PMC3141275

[B19] Depristo MA Banks E Poplin R Garimella KV Maguire JR Hartl C Philippakis AA Del Angel G Rivas MA Hanna M McKenna A Fennell TJ Kernytsky AM Sivachenko AY Cibulskis K Gabriel SB Altshuler D Daly MJ A framework for variation discovery and genotyping using next-generation DNA sequencing data Nat Genet 2011 43 5 491 498 10.1038/ng.806 21478889 PMC3083463

[B20] Qu Y Tan M Kutner MH Random effects models in latent class analysis for evaluating accuracy of diagnostic tests Biometrics 1996 52 3 797 810 10.2307/2533043 8805757

[B21] Menten J Boelaert M Lesaffre E Bayesian latent class models with conditionally dependent diagnostic tests: a case study Stat Med 2008 27 22 4469 4488 10.1002/sim.3317 18551515

[B22] Li H Handsaker B Wysoker A Fennell T Ruan J Homer N Marth G Abecasis G Durbin R 1000 Genome Project Data Processing Subgroup The sequence alignment/map format and SAMtools Bioinformatics 2009 25 16 2078 2079 10.1093/bioinformatics/btp352 19505943 PMC2723002

[B23] Danecek P Auton A Abecasis G Albers CA Banks E Depristo MA Handsaker RE Lunter G Marth GT Sherry ST McVean G Durbin R 1000 Genomes Project Analysis Group The variant call format and VCFtools Bioinformatics 2011 27 15 2156 2158 10.1093/bioinformatics/btr330 21653522 PMC3137218

[B24] Ledergerber C Dessimoz C Base-calling for next-generation sequencing platforms Brief Bioinform 2011 12 5 489 497 10.1093/bib/bbq077 21245079 PMC3178052

[B25] Hui SL Zhou XH Evaluation of diagnostic tests without gold standards Stat Methods Med Res 1998 7 4 354 370 10.1191/096228098671192352 9871952

[B26] Garrett ES Eaton WW Zeger S Methods for evaluating the performance of diagnostic tests in the absence of a gold standard: a latent class model approach Stat Med 2002 21 9 1289 1307 10.1002/sim.1105 12111879

[B27] Pepe MS Janes H Insights into latent class analysis of diagnostic test performance Biostatistics 2007 8 2 474 484 17085745 10.1093/biostatistics/kxl038

[B28] Xu H Craig BA A probit latent class model with general correlation structures for evaluating accuracy of diagnostic tests Biometrics 2009 65 4 1145 1155 10.1111/j.1541-0420.2008.01194.x 19210729

[B29] Roth A Ding J Morin R Crisan A Ha G Giuliany R Bashashati A Hirst M Turashvili G Oloumi A Marra MA Aparicio S Shah SP JointSNVMix: a probabilistic model for accurate detection of somatic mutations in normal/tumour paired next-generation sequencing data Bioinformatics 2012 28 7 907 913 10.1093/bioinformatics/bts053 22285562 PMC3315723

[B30] Larson DE Harris CC Chen K Koboldt DC Abbott TE Dooling DJ Ley TJ Mardis ER Wilson RK Ding L SomaticSniper: identification of somatic point mutations in whole genome sequencing data Bioinformatics 2012 28 3 311 317 10.1093/bioinformatics/btr665 22155872 PMC3268238

[B31] Saunders CT Wong WSW Swamy S Becq J Murray LJ Cheetham RK Strelka: accurate somatic small-variant calling from sequenced tumor-normal sample pairs Bioinformatics 2012 28 14 1811 1817 10.1093/bioinformatics/bts271 22581179

[B32] Cibulskis K Lawrence MS Carter SL Sivachenko A Jaffe D Sougnez C Gabriel S Meyerson M Lander ES Getz G Sensitive detection of somatic point mutations in impure and heterogeneous cancer samples Nat Biotechnol 2013 31 3 213 219 10.1038/nbt.2514 23396013 PMC3833702

[B33] Löwer M Renard BY de Graaf J Wagner Paret C Kneip C Türeci O Diken M Britten C Kreiter S Koslowski M Castle JC Sahin U Confidence-based somatic mutation evaluation and prioritization PLoS Comput Biol 2012 8 9 e1002714 10.1371/journal.pcbi.1002714 23028300 PMC3459886

