# Supplementary material for: Comparing somatic mutation-callers: beyond Venn diagrams
Source: BMC Bioinformatics. 2013 Jun 10;14:189. doi: 10.1186/1471-2105-14-189 (PMC3702398; doi:10.1186/1471-2105-14-189)
Supplement: Additional file 1 — Supplementary tables and figures. [file 1471-2105-14-189-S1.pdf]

Supplementary tables and figures

Comparing somatic mutation-callers: beyond Venn diagrams

Su Yeon Kim[1*] and Terence P. Speed[1,2*]

[1]Department of Statistics, University of California at Berkeley, 367 Evans Hall, Berkeley, CA 94720 USA

[2]Walter and Eliza Hall Institute of Medical Research, Parkville, Victoria, Australia

[*] Corresponding authors

Email addresses:

SYK: skim@stat.berkeley.edu
TPS: terry@stat.berkeley.edu

Table S1: Summary of the analyzed datasets. We have analyzed the mutation-calling outputs in two datasets from the TCGA benchmark studies: the LUSC and READ datasets. We have also created an ‘evaluation set’ for method development (for details, see Methods).

	LUSC dataset	READ dataset	Evaluation set
Mutation/variant calling algorithms	Caller A, B, C, D	Caller H, I, J	GATK UnifiedGenotyper
Sources of data	16 LUSC patients	6 READ patients	39 LUSC patients
Regions in which mutations/variants are called	exome	exome	76 genes
Sequence data used for mutation/variant calling	Illumina exome-seq tumor-normal pairs	SOLiD exome-seq tumor-normal pairs	Illumina tumor exome-seq
Gold-standard validation data	Deep-sequencing tumor-normal pairs on 76 genes	Validation information for 721 mutations	Deep-sequencing tumor-normal pairs on 76 genes
Additional data 1	RNA-seq data for tumor samples	RNA-seq data for tumor samples	RNA-seq data for tumor samples
Additional data 2		Illumina exome-seq tumor-normal pairs	

Table S2: Counts of the mutations called based on the SOLiD exome-seq pairs for three READ patients. The variants included in any mutation output (VCF) file were divided into detection status of the three callers (Caller H, I, and J). Each mutation was classified into five groups. It is 'nonMAF' if the mutation found in the benchmark data but did not appear in the TCGA colon working group mutation file (MAF as of Nov 8, 2011) nor validated by the 454 technology. Otherwise, it was classified as 'unknown' (in the MAF file but not validated), 'wildtype' (no variant allele found in either the tumor or the normal sample), 'germline' (variant allele found in both the tumor and the normal sample), and 'somatic' (the variant allele is found in the tumor sample but not in the normal).

READ-2	nonMAF	unknown	wildtype	germline	somatic
Caller H only	190	0	0	0	0
Caller I only	8	0	0	0	0
Caller H and Caller I	5	0	0	0	0
Caller J only	55	2	1	3	2
Caller H and Caller J	1	1	1	0	4
Caller I and Caller J	3	2	2	0	5
All centers	1	11	0	0	42

READ-3	nonMAF	unknown	wildtype	germline	somatic
Caller H only	128	0	0	0	0
Caller I only	58	0	0	0	0
Caller H and Caller I	14	0	0	0	0
Caller J only	62	38	168	1	18
Caller H and Caller J	1	0	6	1	7
Caller I and Caller J	0	3	51	0	24
All centers	2	0	31	1	34

READ-4	nonMAF	unknown	wildtype	germline	somatic
Caller H only	113	11	1	0	1
Caller I only	31	0	0	0	0
Caller H and Caller I	0	1	0	0	0
Caller J only	91	0	56	0	0
Caller H and Caller J	3	0	1	0	8
Caller I and Caller J	2	0	8	0	2
All centers	1	0	2	0	34

Table S3: Observed and the fitted counts based on the latent class models used for the benchmark data, either only using the mutations within the 76 genes or the whole exome. For the set using the 76 genes, the model assumes the conditional independence among the callers. For the whole exome data, a latent class model with random effects was used.

Mutation set	Observed_76genes	Fitted_76genes	Observed_exome	Fitted_exome
None	4301	4300.99	22,461	22,455.80
Caller A only	7	7.01	476	483.91
Caller B only	3	3.00	58	56.47
Caller C only	2	2.00	322	324.56
Caller D only	11	11.02	415	419.22
Caller A and Caller B	0	0.00	14	36.99
Caller A and Caller C	6	2.67	292	211.60
Caller A and Caller D	5	2.68	164	127.92
Caller B and Caller C	2	1.54	78	103.28
Caller B and Caller D	2	1.54	74	57.05
Caller C and Caller D	5	5.78	241	326.26
All but Caller D	3	6.08	208	236.91
All but Caller C	4	6.06	104	129.06
All but Caller B	18	22.85	716	737.92
All but Caller A	13	13.14	466	372.87
All centers	57	51.94	1,667	1,676.17

A.

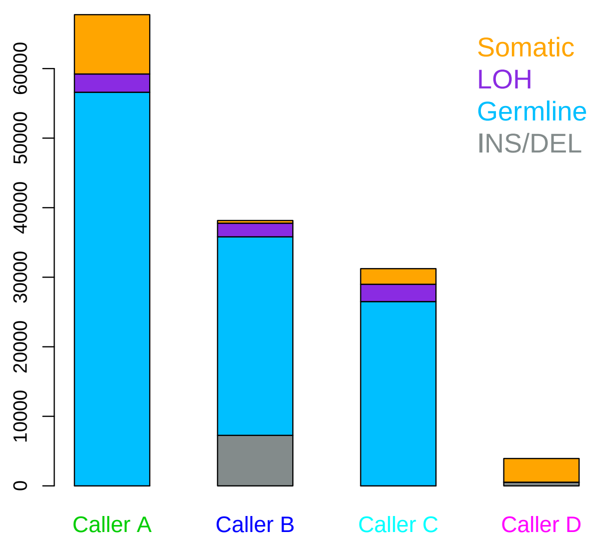

B.

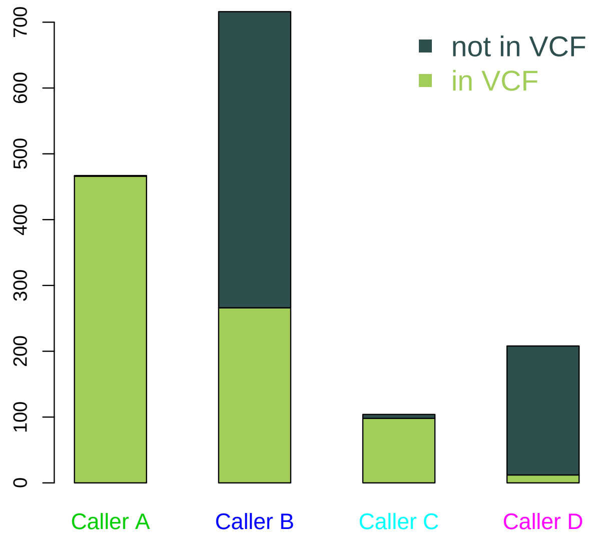

Figure S1: Discrepancies in the reported variants in VCF files. A. The number and the types of variants reported in each of the four VCF files generated from a single tumor-normal LUSC seq-pair. Other seq-pairs generate a similar graph. B. Among the mutations that were missed by a single caller, the number of mutations that were found or not found in the VCF file of the caller that missed those mutations. Mutations were aggregated over the data from 16 LUSC patients used for the benchmark study.

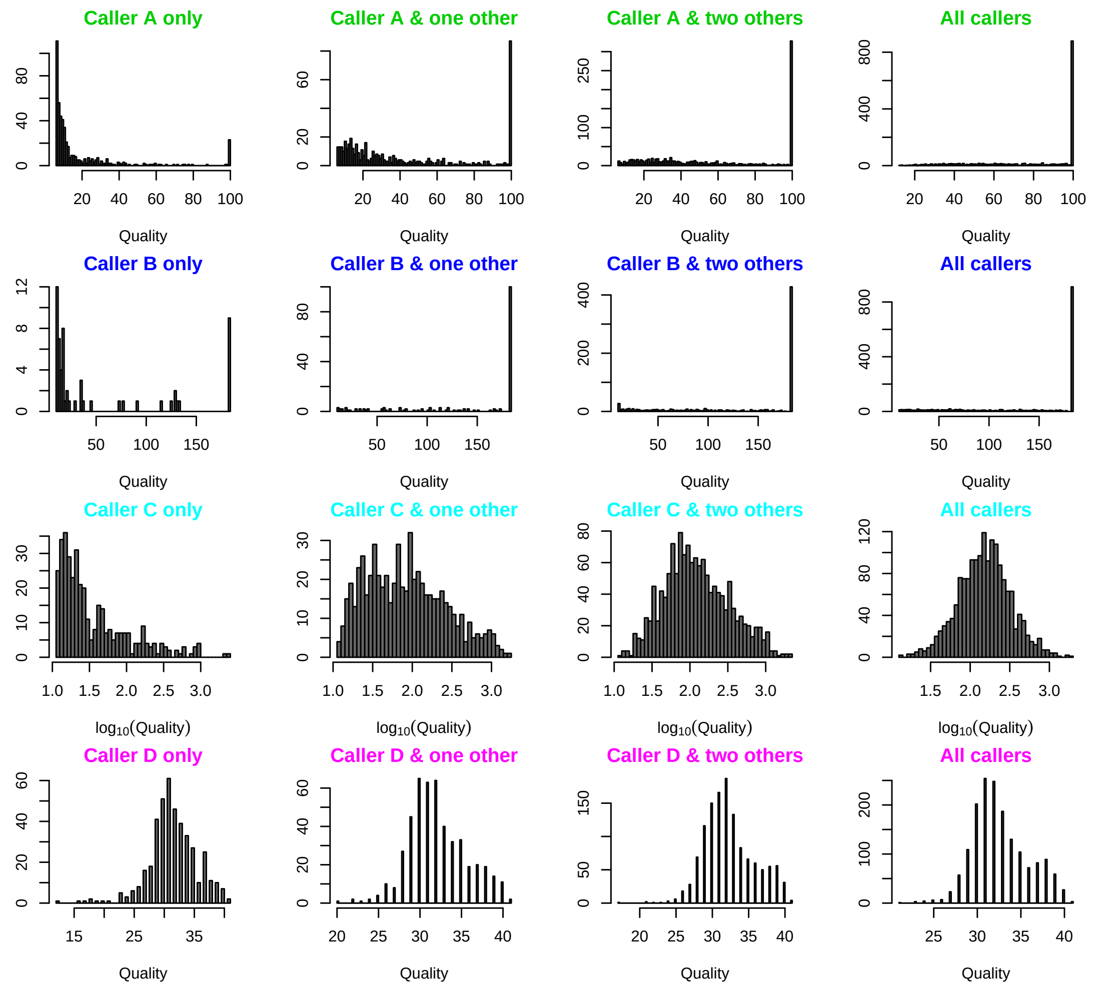

Figure S2: Distribution of mutation quality score reported in VCF files. Within each caller's output (row), mutations from 16 LUSC patients are stratified by the number of callers that detected the mutations (column).

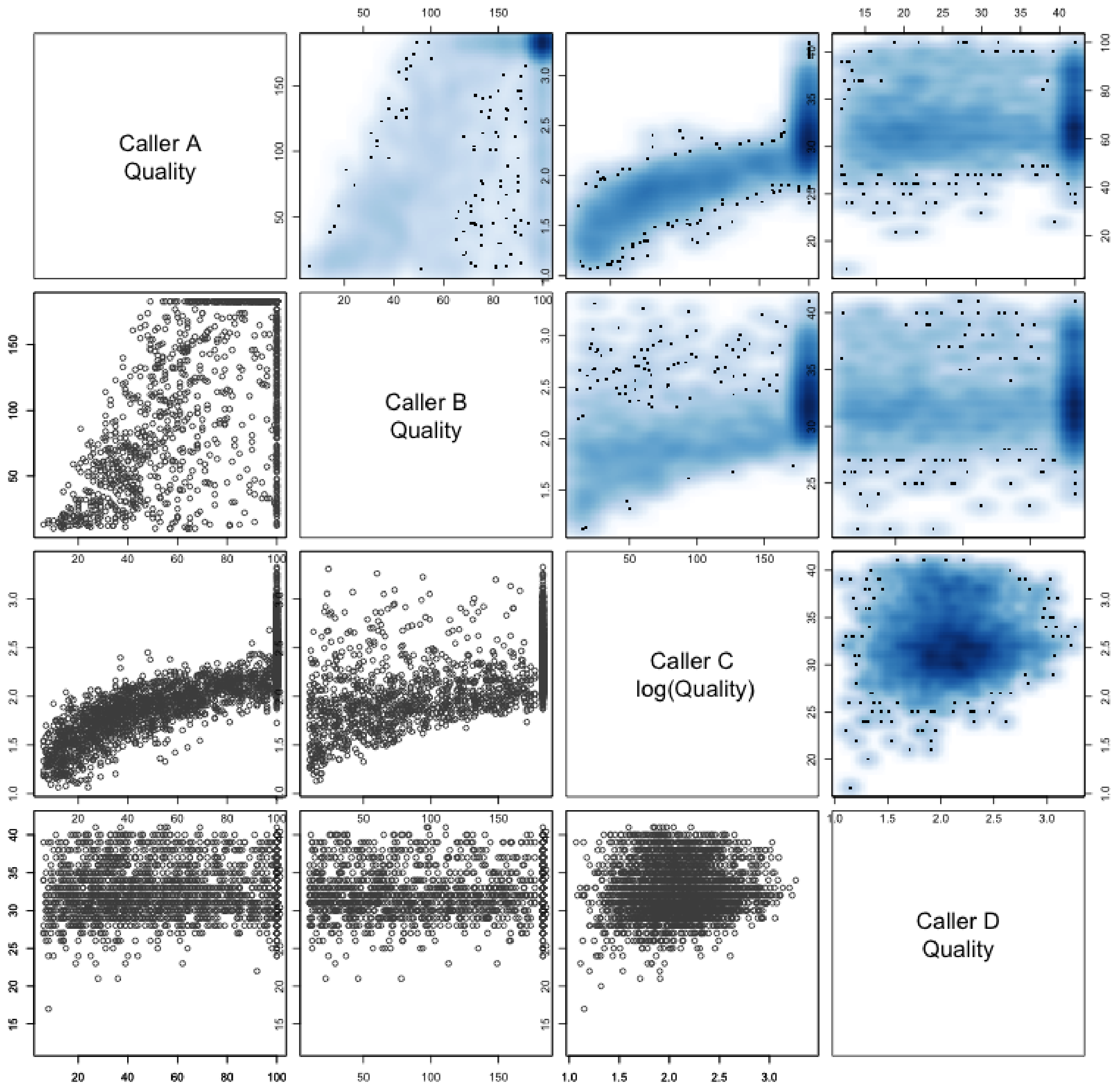

Figure S3: Pairwise comparison of mutation quality scores reported in the VCF files. Mutations that were detected by all callers from 16 LUSC patients are plotted.

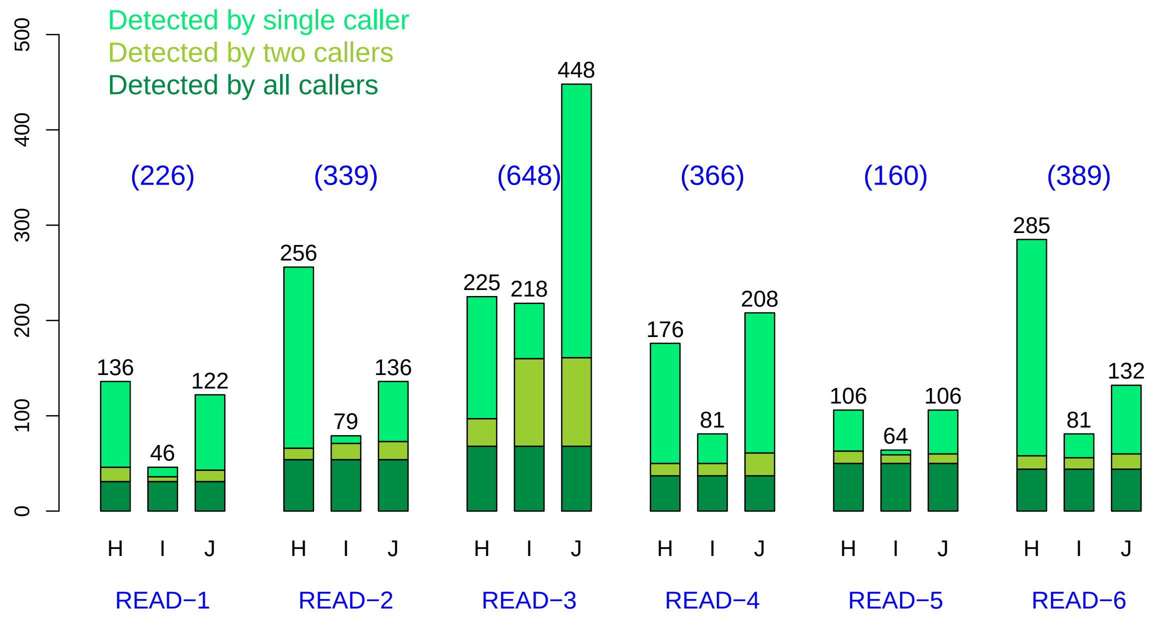

Figure S4: Counts of the mutations across 6 READ patients. The mutations detected by each caller (H, I, or J character specified in the horizontal axis) are stratified by the number of callers detecting those mutations. The number right on top of each bar is the number of mutations called by each caller. The number within the parentheses above the middle bar for each READ patient is the total number of mutations detected by one or more of the three callers.

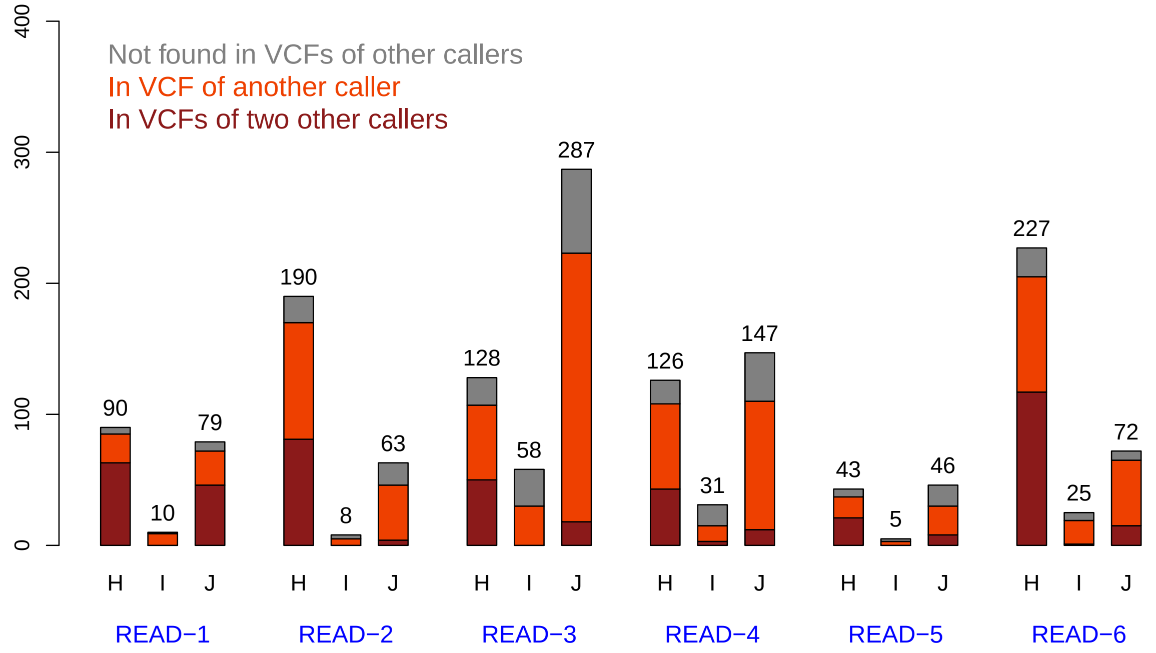

Figure S5: Counts of mutations detected by a single caller only (specified in the horizontal axis: H, I, or J) that are stratified by the number of *other* callers reporting the mutations in their raw mutation-outputs (VCF files). Mutations are from 6 READ patients.

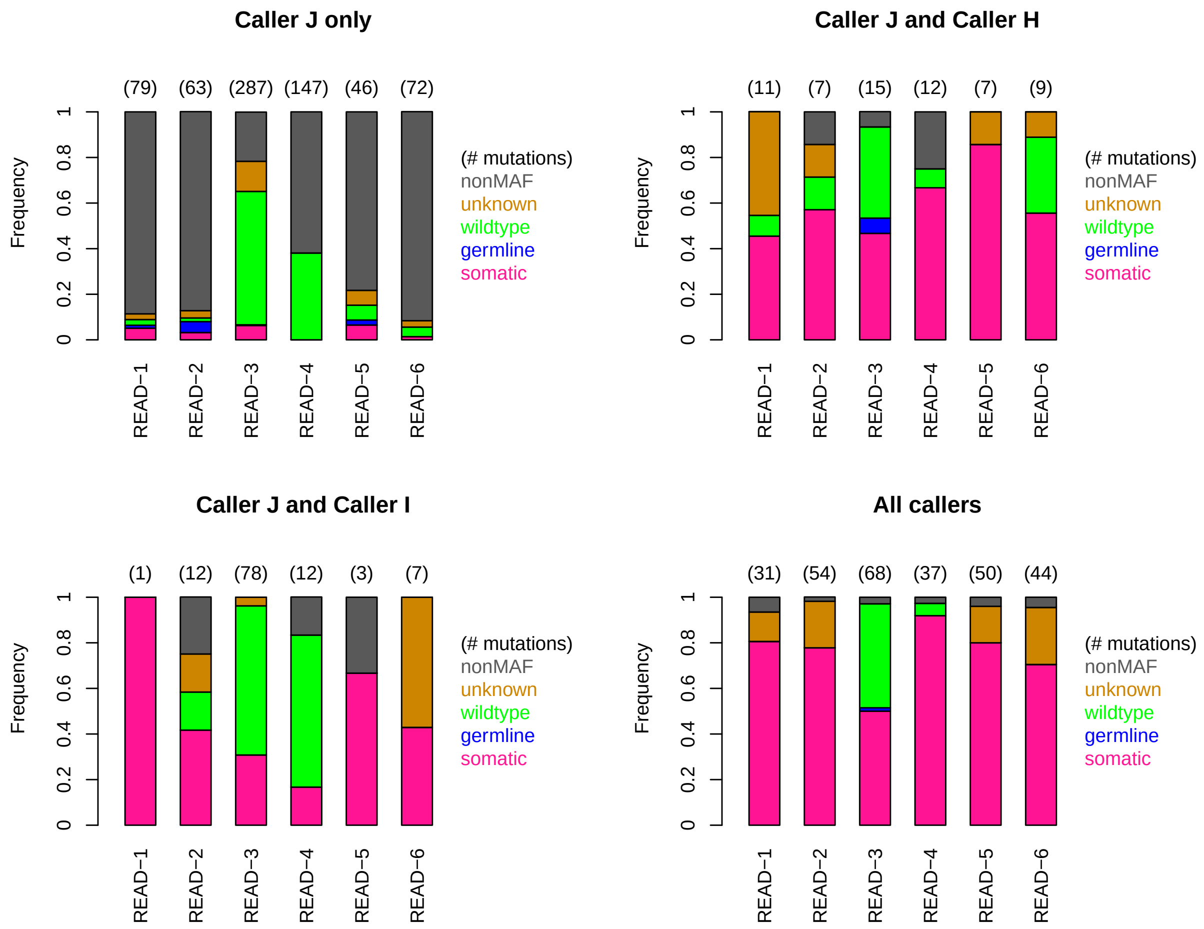

Figure S6: Validation results for four mutation categories across 6 READ patients. For each mutation category (defined based on the call status), the corresponding panel shows the counts of the mutations (number within the parentheses on top of each bar) and the fraction of mutations in each validation group, across 6 READ patients. For the definition of the validation groups, see the caption of Table S2. Note that since only the mutations detected by Caller J were attempted to be validated, we only present mutations categories that ensure the detection of Caller J.

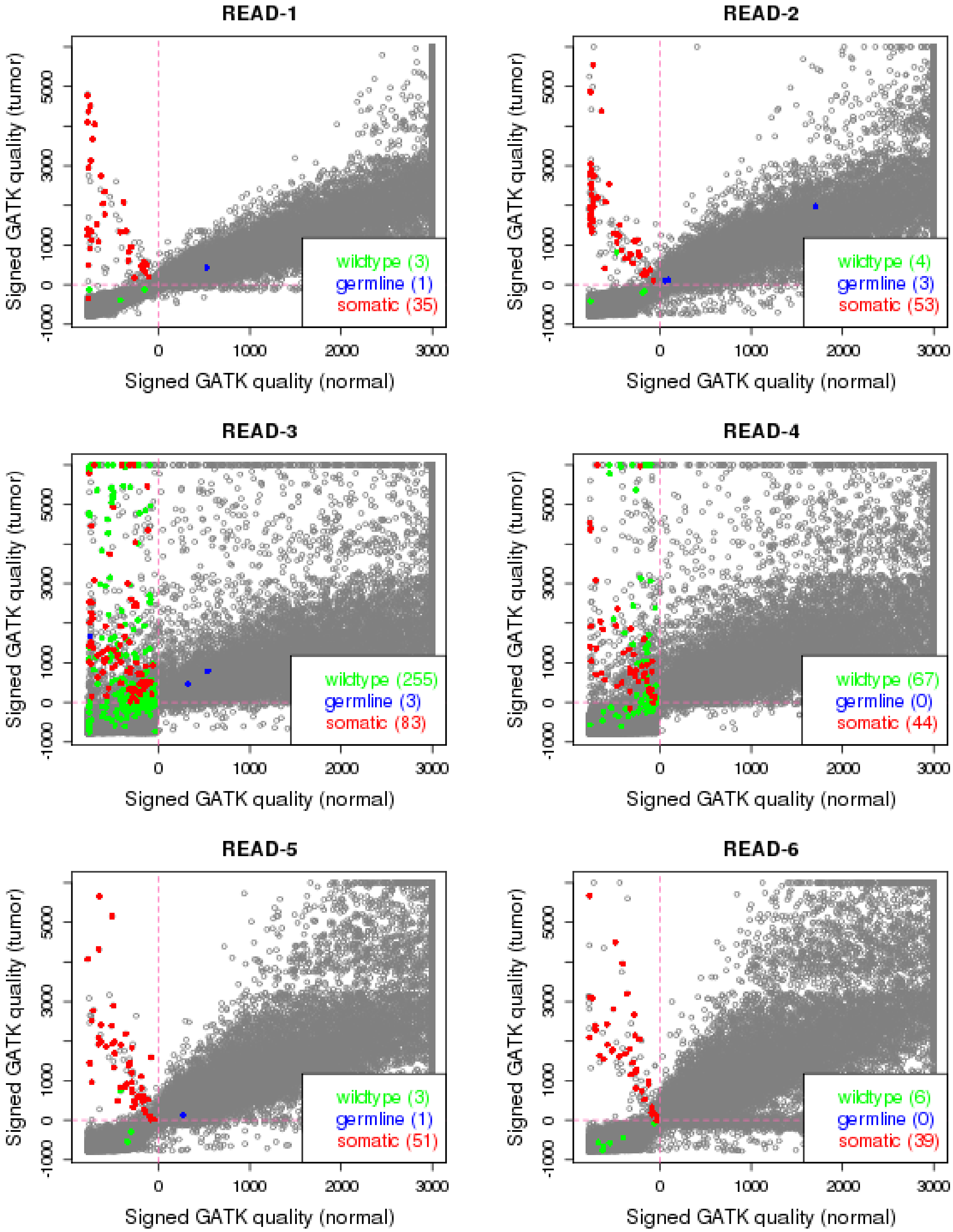

Figure S7: Distribution of signed GATK quality scores of tumor-normal exome-seq pairs from 6 READ patients. For each variant reported in any of the VCF files, the GATK quality scores were obtained using Illumina exome-seq pairs. (For more details, see the caption of Figure 4.) The variants validated by 454 technology are indicated with color: red (somatic), blue (germline), and green (wildtype).

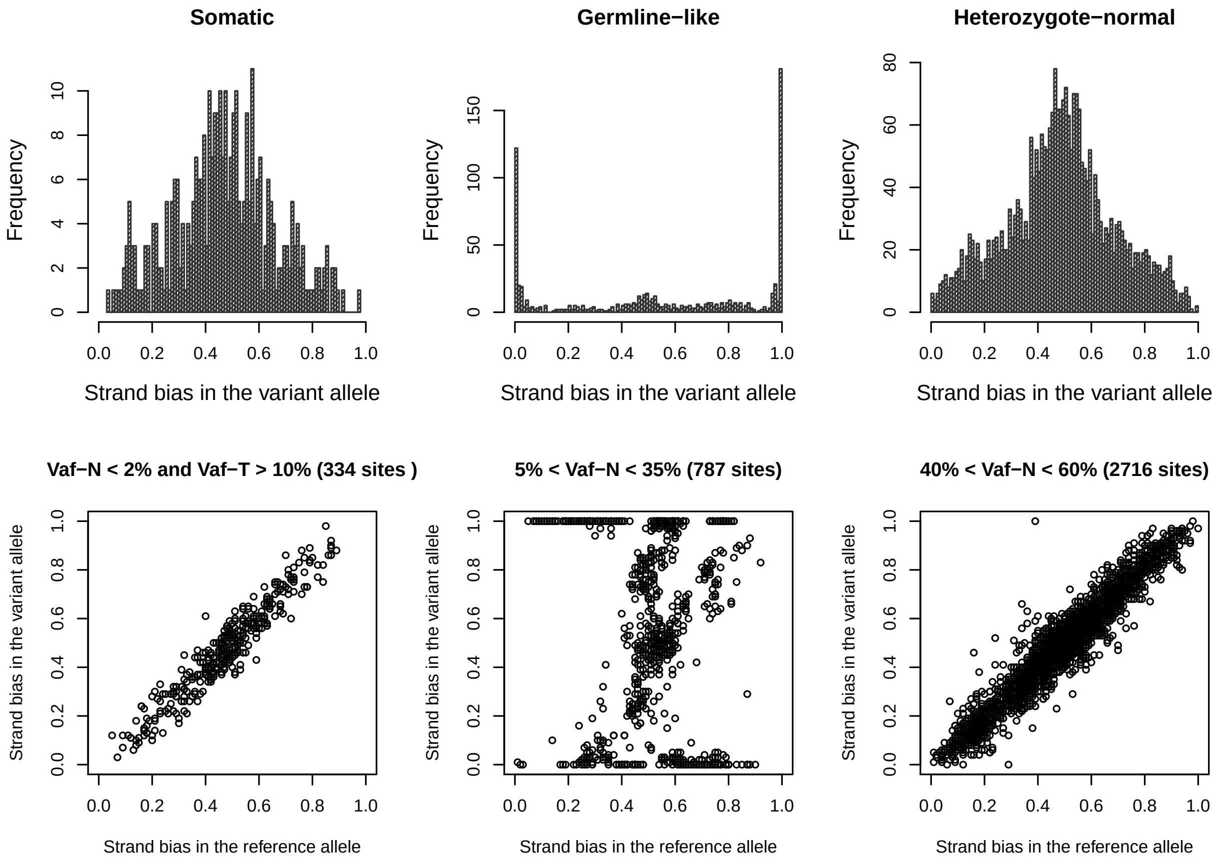

Figure S8: Strand bias pattern in the tumor deep-sequence data used for the evaluation datset, across three variant classes defined based on the tumor and normal vafs: (1) the normal vaf is $< 2\%$ and the tumor vaf is $> 10\%$ ('Somatic'; left column), (2) the normal vaf is between 5% and 35% (named as 'Germline-like' since the tumor vaf is highly correlated with the normal vaf for almost all the variants in this class; middle column), and (3) the normal vaf is between 40% and 60% (named as 'Heterozygote-normal' since the vaf range implies heterozygous genotype in the normal sample; right column). The strand bias of an allele is measured by the fraction of the reads on the forward strand among the reads carrying the allele. Histograms using the strand bias of the variant allele are shown in the upper row. Scatter plots showing the strand bias of the variant allele (y-axis) vs the reference allele (x-axis) are shown in the lower row.

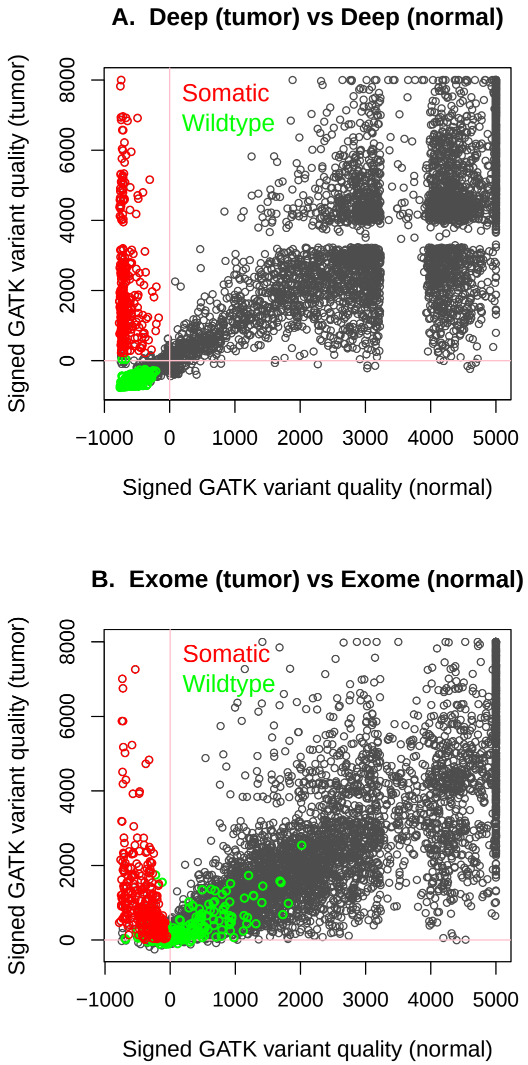

Figure S9: Scatter plots of the *signed* GATK variant quality scores obtained using the tumor-normal deep-seq pairs (upper) and the tumor-normal exome-seq pairs (lower). Each point is a variant detected in the tumor exome-seq using the `GATK UnifiedGenotyper`. For more details, see the caption of Figure 5, which utilizes the same variants. For each variant site, we obtain the GATK variant quality score using a tumor sequence (y-axis) and the normal sequence (x-axis). When no variant is detected, we flipped the sign.

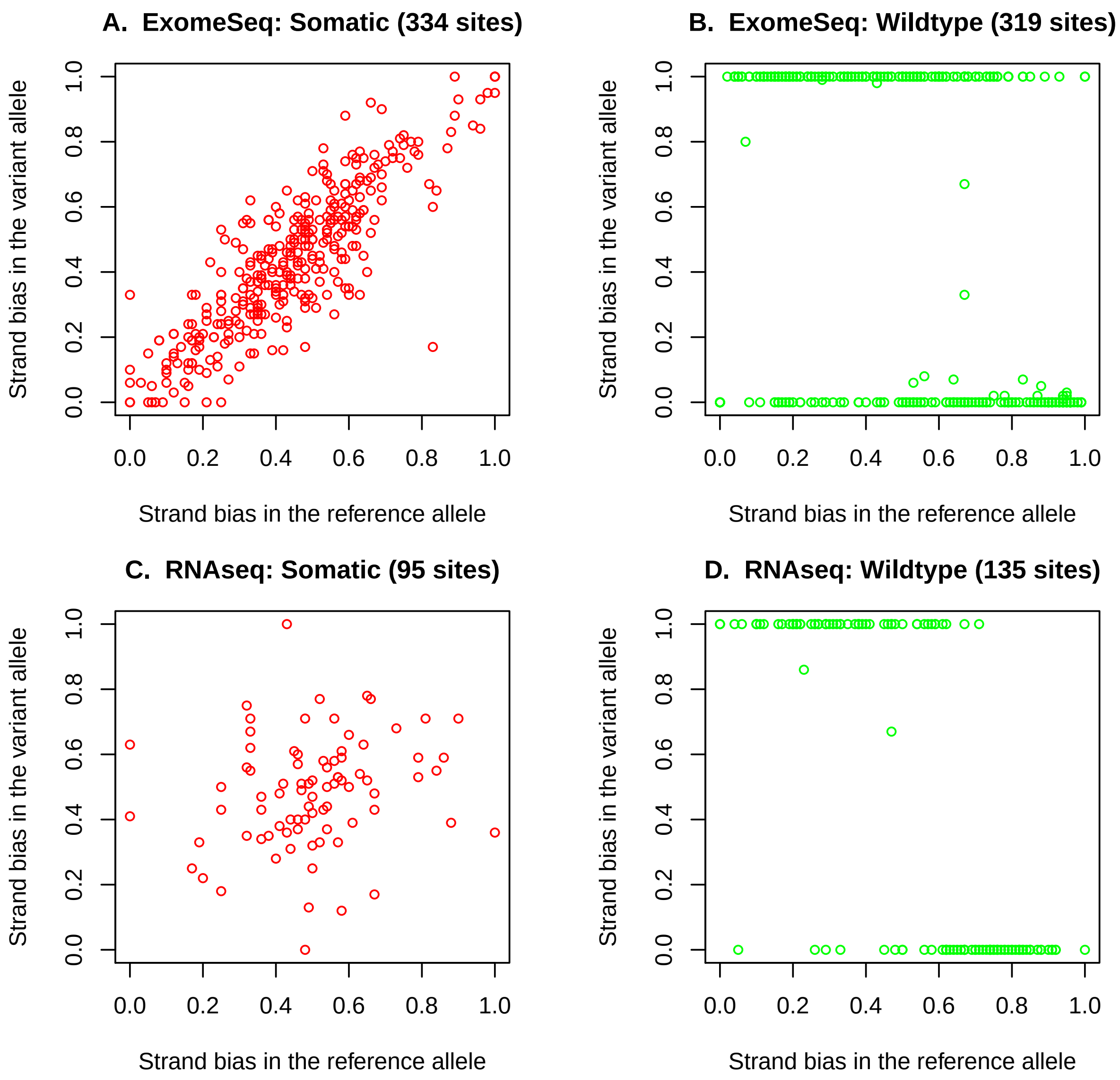

Figure S10: Comparison of strand bias pattern between 'somatic' mutations and 'wildtypes', in the tumor exome-seq (upper) and RNA-seq (lower). (For detailed definition of the two types of variants, see the caption of Figure 5.) At each variant site, for each allele of interest, we measured the strength of strand bias by the fraction of reads on the forward strand among the reads carrying the allele. In the tumor exome-seq, somatic sites show a high correlation between the strand bias of the variant allele and the reference allele (upper left), while for wildtypes, the strand bias of the variant allele tends to be very extreme, i.e., near zero or one (upper right).

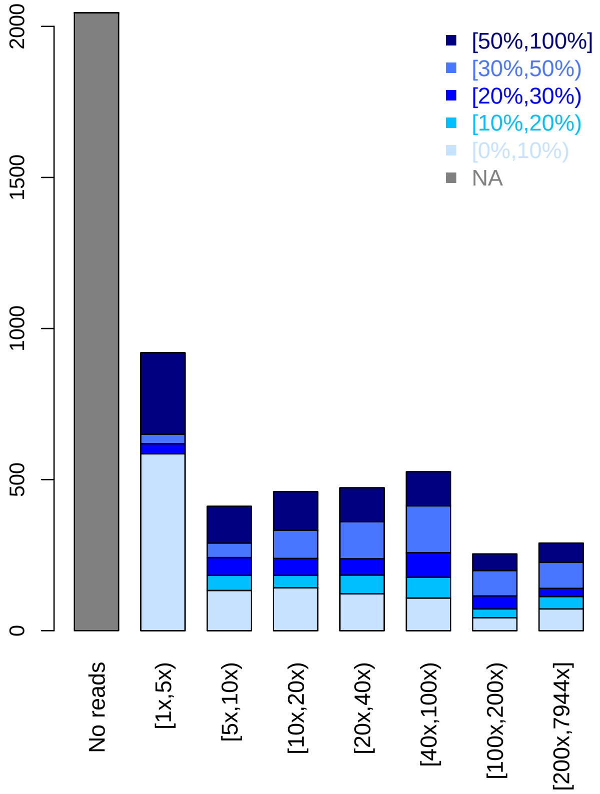

Figure S11: Distribution of the coverage (horizontal) and the variant allele fraction (vertical) in the RNA-seq data. Mutations in the whole exome benchmark data from 16 LUSC patients (5,380 sites) were used.

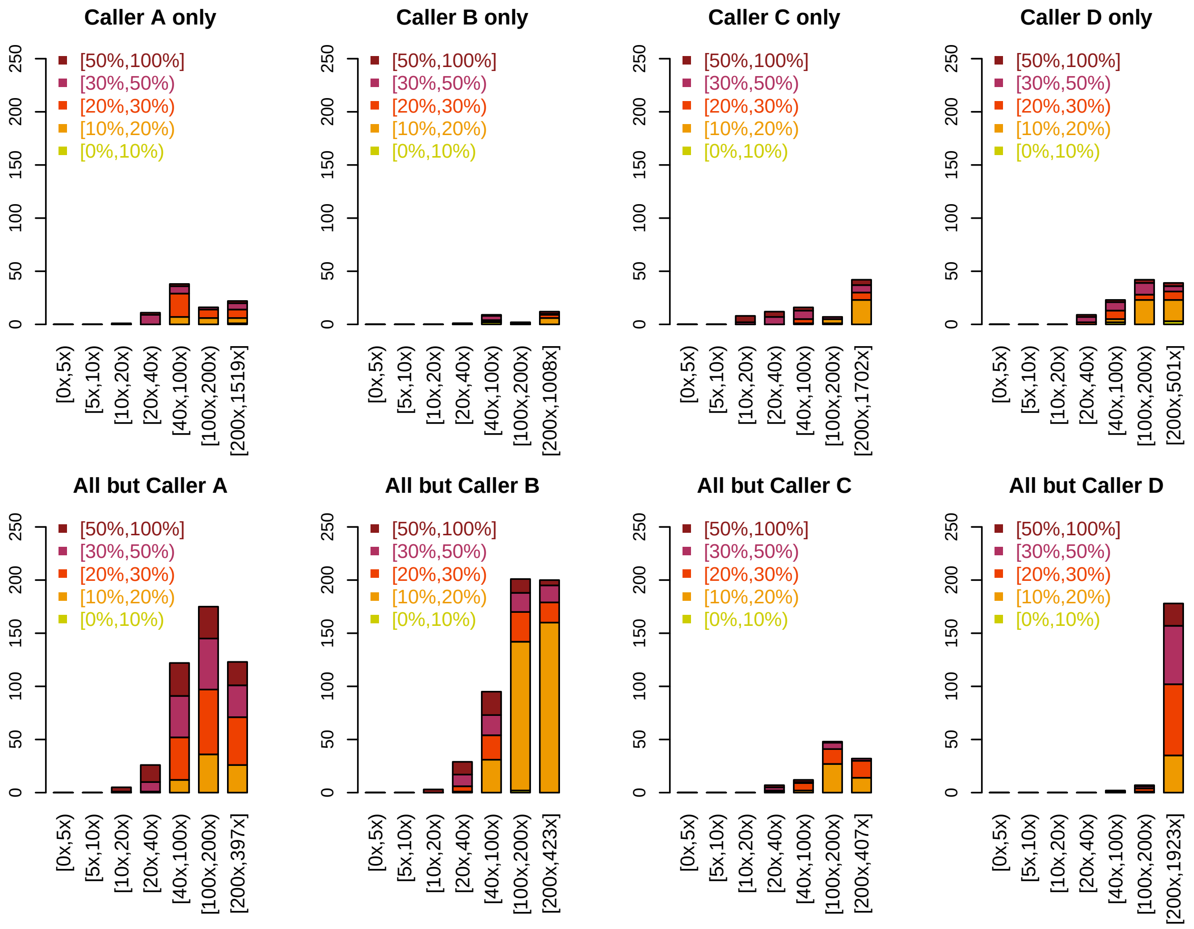

Figure S12: Distribution of the coverage (horizontal) and the variant allele fraction (vertical) of the mutations validated as positive by the pseudo-validation method in the tumor exome-seq data.

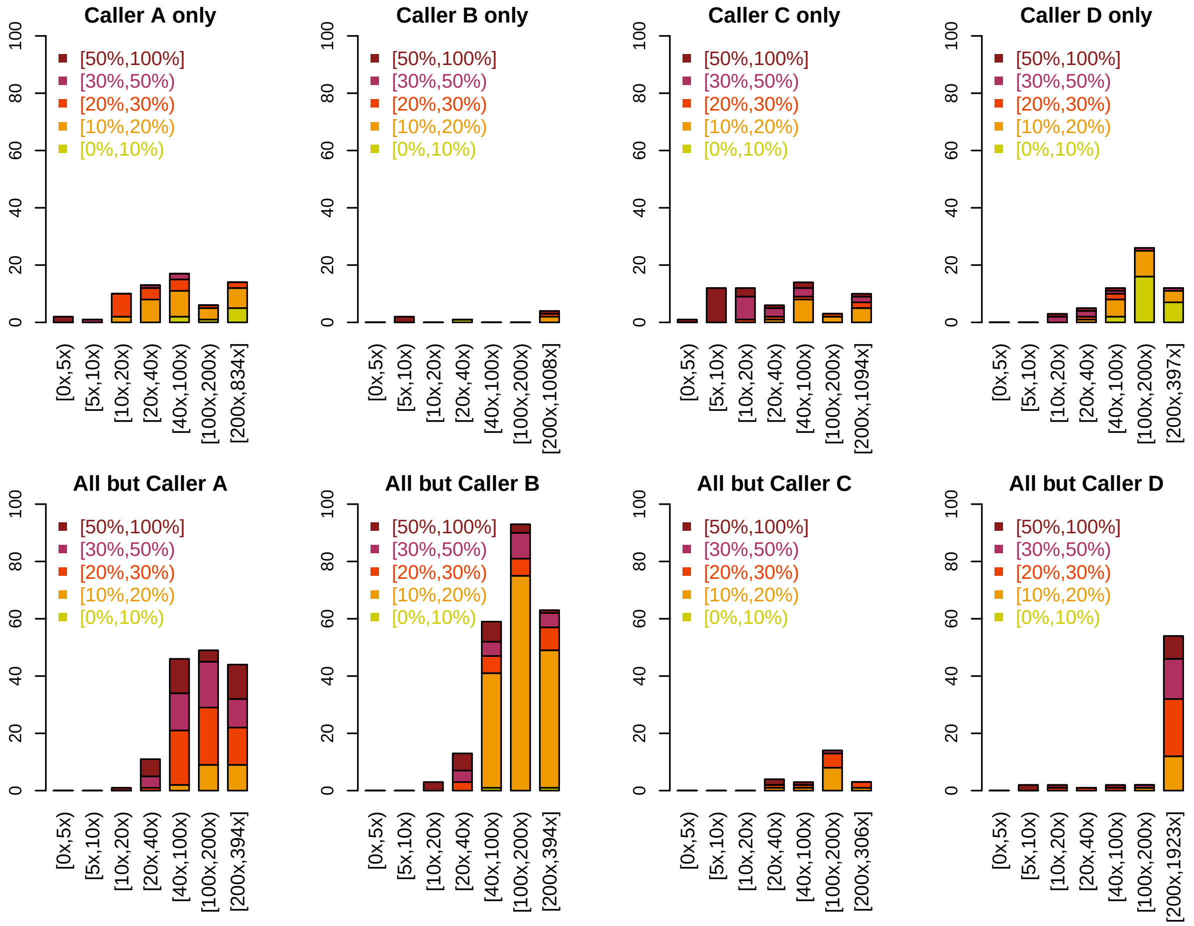

Figure S13: Distribution of the coverage (horizontal) and the variant allele fraction (vertical) of the mutations validated as positive by the RNA-seq validation method in the tumor exome-seq data.

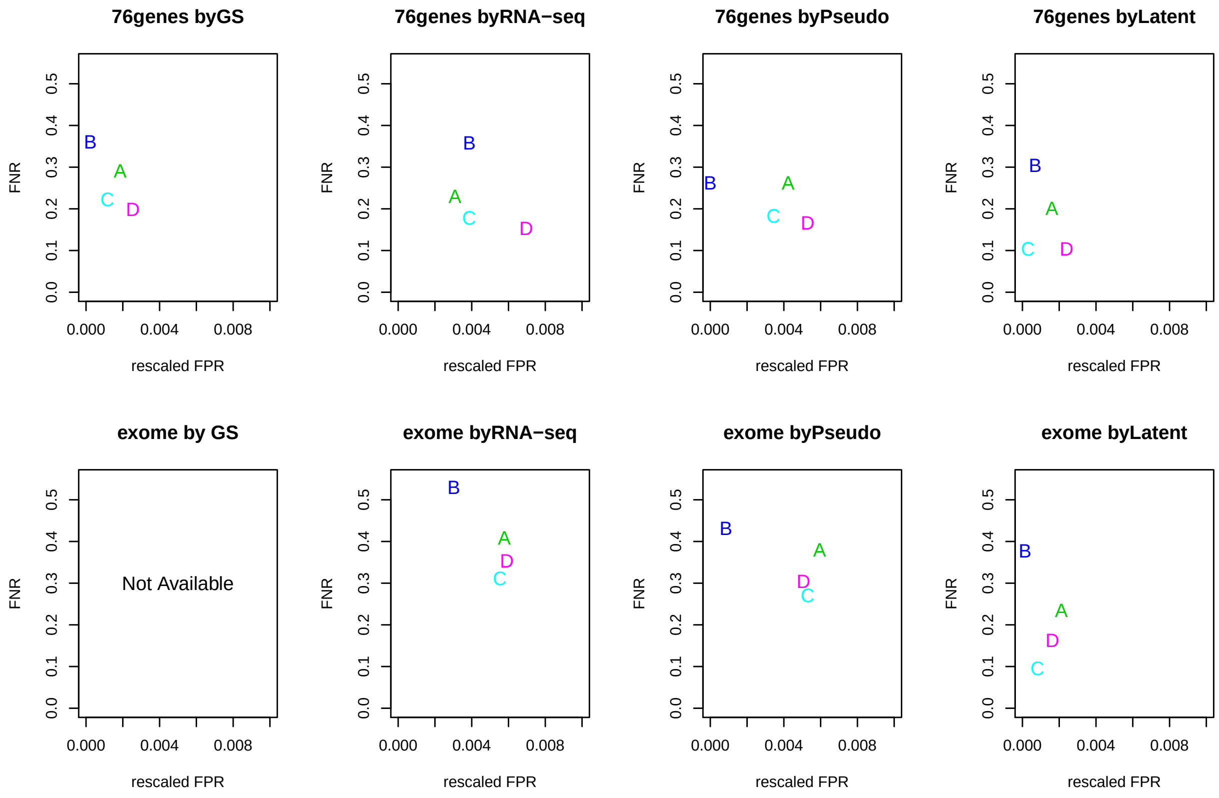

Figure S14: False negative vs (re-scaled) false positive rates estimated using two datasets (row) by four validation methods (column). For detailed description, see the caption of Figure 7.
